# Supplementary material for: Paf1 Has Distinct Roles in Transcription Elongation and Differential Transcript Fate
Source: Mol Cell. 2017 Feb 16;65(4):685–698.e8. doi: 10.1016/j.molcel.2017.01.006 (PMC5316414; doi:10.1016/j.molcel.2017.01.006)
Supplement: Document S1. Figures S1–S5 and Table S5 [file mmc1.pdf]

Molecular Cell, Volume *65*

Supplemental Information

Paf1 Has Distinct Roles in Transcription Elongation and Differential Transcript Fate

Harry Fischl, Françoise S. Howe, Andre Furger, and Jane Mellor

SUPPLEMENTAL INFORMATION

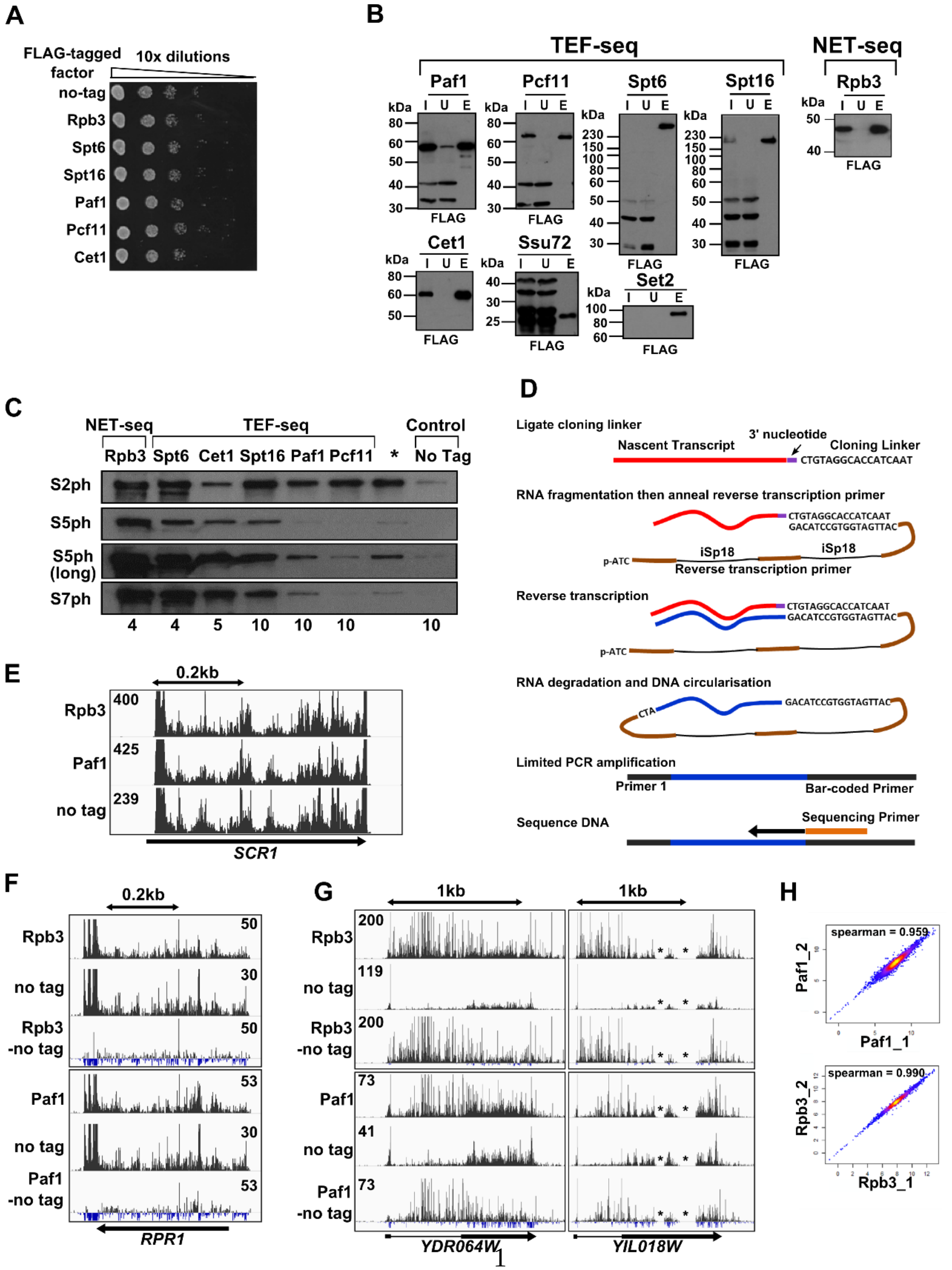

Figure S1, **Related to Figure 1. TEF-seq library preparation and sequencing. A** FLAG-tagging TEFs or Rpb3 does not influence growth rate. Drop plates showing serial 10-fold dilution of strains indicated on rich medium (YPD). **B** Western blots showing IP of FLAG-tagged factors. Large proteins such as Spt6 and Spt16 do not reliably produce a signal in total cell extracts at the concentrations used. I, input; U, unbound; E, eluted with FLAG peptide. **C** Autoradiographs of Western blots showing CTD phosphorylation levels for Ser2, Ser5 (two exposure lengths) and Ser7 on Rpb1 co-IPed with FLAG-tagged Rpb3 (NET-seq), FLAG-tagged TEFs (TEF-seq) or an untagged IP (Control, no-tag). Volume (µl) of each IP used is shown below the autoradiographs. * is an unrelated sample. **D** Schematic of library preparation and sequencing. **E** Background signal that is not specific to the tagged factor, resulting in reads over Pol3-transcribed *SCR1* in raw data from Rpb3 NET-seq, Paf1 TEF-seq and a no-tag control IP (no tag). The level of the signal at *SCR1* was used to remove this background signal from experimental samples genome-wide. **F-G** Images from IGV showing NET-seq reads over Pol2-transcribed *RPS13* and *RPL2B* and Pol3-transcribed *RPR1* for Rpb3-FLAG compared to TEF-seq reads for Paf1-FLAG before (top track) and after (bottom track) subtraction of the no-tag reads from a non-tagged control IP (middle track). * marks gaps in the profiles resulting from discarding non-uniquely aligned reads. Pre-subtraction tracks are scaled relative to the no-tag track according to the ratio of reads in each aligning to the *SCR1* gene. No-tag subtracted tracks are on the same scales as their pre-subtraction tracks. The loss of signal after no-tag subtraction in both the Pol2-specific transcription component Paf1 and Rpb3 tracks at Pol3-transcribed *RPR1* demonstrates that the non-specific signal is effectively removed. **H** Scatter plots showing the high similarity between replicates for NET-seq for Pol2 (Rpb3) and TEF-seq for Paf1. For each replicate, after subtraction of the no-tag control signal, reads mapping to each mRNA gene, from the TSS to the PAS were correlated using spearman correlation coefficients after applying a regularised log (rlog) transformation (Love et al., 2014). Scatter plots are colour-coded from low density (blue) to high density (yellow).(Related to Figure 1).

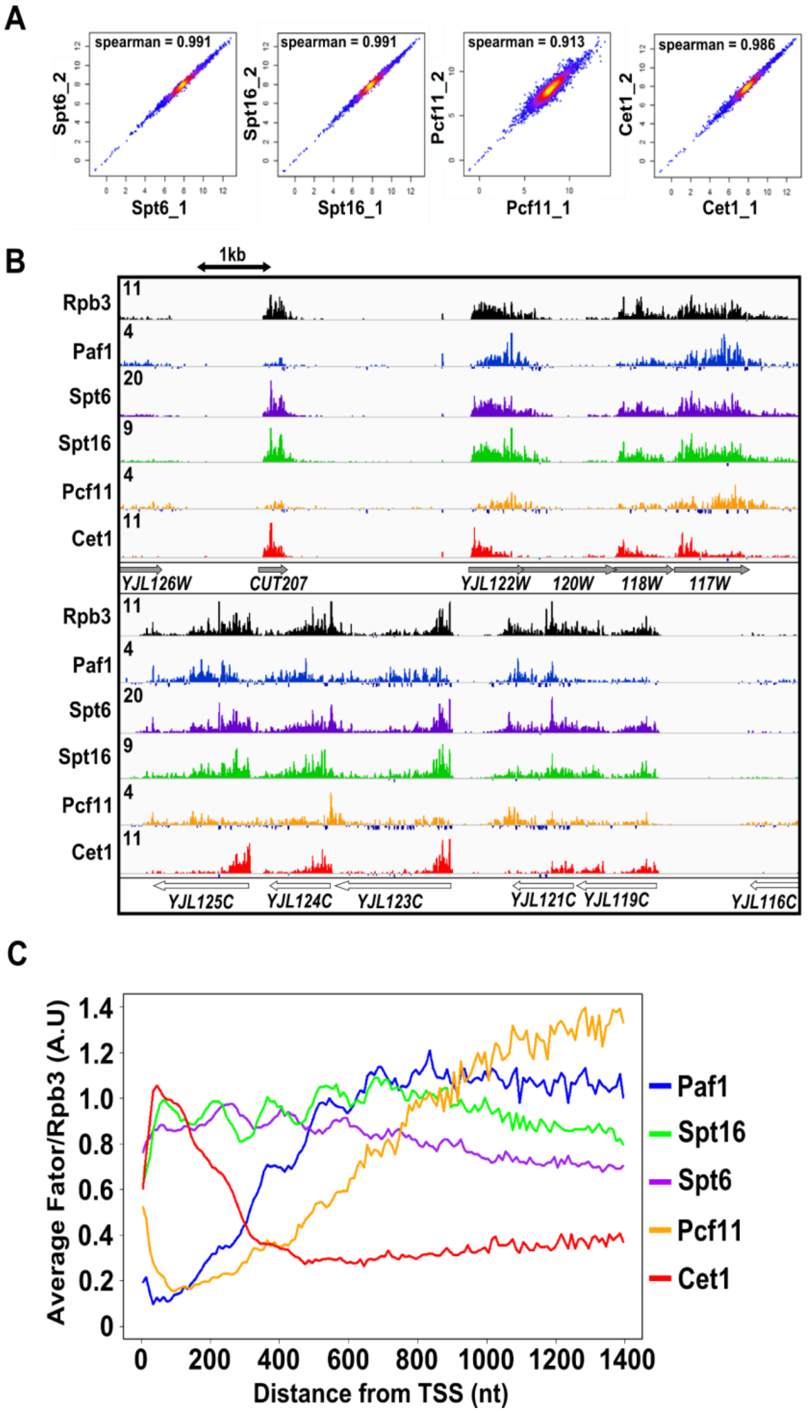
A
spearman = 0.991
Spt6_2
Spt6_1
spearman = 0.991
Spt16_2
Spt16_1
spearman = 0.913
Pcf11_2
Pcf11_1
spearman = 0.986
Cet1_2
Cet1_1
B
1kb
Rpb3
Paf1
Spt6
Spt16
Pcf11
Cet1
YJL126W
CUT207
YJL122W
120W
118W
117W
YJL125C
YJL124C
YJL123C
YJL121C
YJL119C
YJL116C
C
Average Fator/Rpb3 (A.U)
Distance from TSS (nt)
Paf1
Spt16
Spt6
Pcf11
Cet1

Figure S2, Related to Figures 2 and 3. TEF-seq for Spt6, Spt16 and Pcf11. **A** Scatter plots showing the high similarity between replicates for TEF-seq for Spt6, Spt16, Pcf11 and Cet1. For each replicate, after subtraction of the no-tag control signal, reads mapping to each mRNA gene, from the TSS to the PAS were correlated using spearman correlation coefficients after applying a regularised log (rlog) transformation (Love et al., 2014). Scatter plots are colour-coded from low density (blue) to high density (yellow). **B** Examples of Rpb3 NET-seq and Paf1, Spt6, Spt16, Pcf11 and Cet1 TEF-seq profiles on individual genes displayed in IGV (see Figure 1 for details). **C** Rpb3-normalized TEF metagene profiles for Paf1, Spt6, Spt16, Pcf11 and Cet1 from the TSS to +1400nt averaged across mRNA genes. See Figure 1**C** for details.

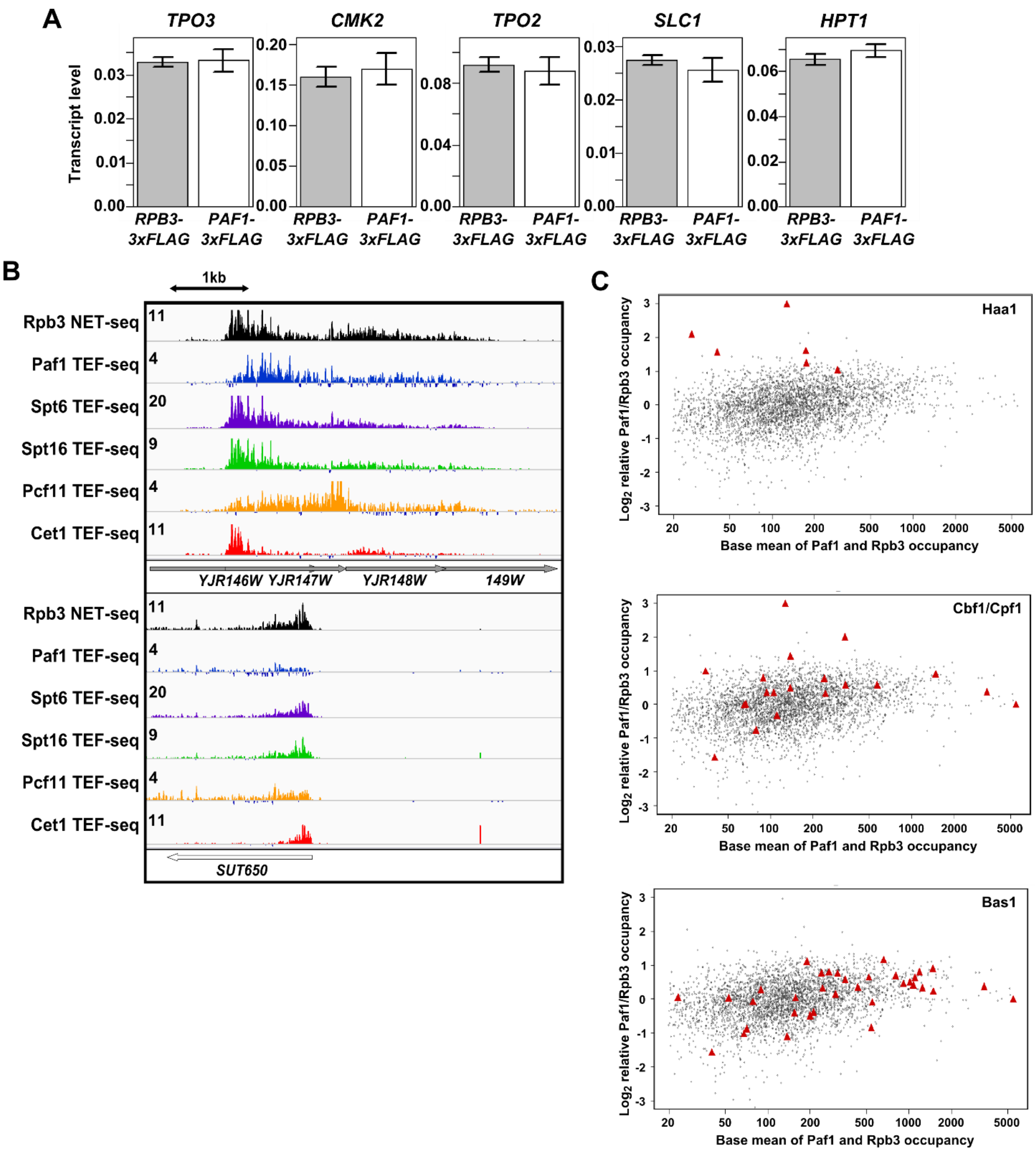
A
TPO3
CMK2
TPO2
SLC1
HPT1
Transcript level
RPB3-3xFLAG
PAF1-3xFLAG
B
1kb
Rpb3 NET-seq
Paf1 TEF-seq
Spt6 TEF-seq
Spt16 TEF-seq
Pcf11 TEF-seq
Cet1 TEF-seq
YJR146W YJR147W
YJR148W
149W
SUT650
C
Haa1
Cbf1/Cpf1
Bas1
Log2 relative Paf1/Rpb3 occupancy
Base mean of Paf1 and Rpb3 occupancy

Figure S3, Related to Figure 3. Selective enrichment and depletion for Paf1 on Pol2. A No difference in RNA levels between *PAF1-3xFLAG* and *RPB3-3xFLAG* strains. Bar charts showing the average level of transcripts encoded by Paf1-enriched (*TPO3*, *CMK2*, *TPO2*), Paf1-depleted (*SLC1*) and no significant difference in Paf1 (*HPT1*) genes in *PAF1-3xFLAG* and *RPB3-3xFLAG* strains. Transcript levels, measured by RT-qPCR, are normalized to the level of the *ADH1* transcript in each sample. Error bars show the S.E.M.; n=5. **B** Examples of Rpb3 NET-seq and Paf1, Spt6, Spt16, Pcf11 and Cet1 TEF-seq profiles on individual genes displayed in IGV (see Figure 1 for details). The Paf1 TEF-seq signal is lower relative to the Rpb3 NET-seq signal on the lncRNA gene *SUT650* compared to the mRNA genes *YJR147W* and *YJR148W.* **C** Differential Paf1 occupancy at mRNA genes (grey circles) shown as a scatter plot of the base mean occupancy against the $\log_2$ relative Paf1 to Rpb3 occupancy ratio for each gene. Red triangles show genes regulated by the transcription factors Haa1 (top); Cbf1/Cpf1 (middle) or Bas1 (bottom). Six genes (*YER130C*/*COM2*, *YIR035C*, *YPR156C*/*TPO3*, *YPR157W*/*TDA6*, *YGR138C*/*TPO2, YBR054W/YRO2*) out of 10 that encode Haa1- and acid stress-responsive membrane proteins are shown to be significantly Paf1-enriched. The remaining four genes in this group were too lowly transcribed to be selected for differential occupancy analysis (Keller et al., 2001). Other Paf1-enriched genes, such as *YOL016C*/*CMK2* have also been shown to be regulated by Haa1 (Fernandes et al., 2005; Hu et al., 2007). Like many transcription factors, Bas1 does not show enrichment for regulation of Paf1-enriched genes.

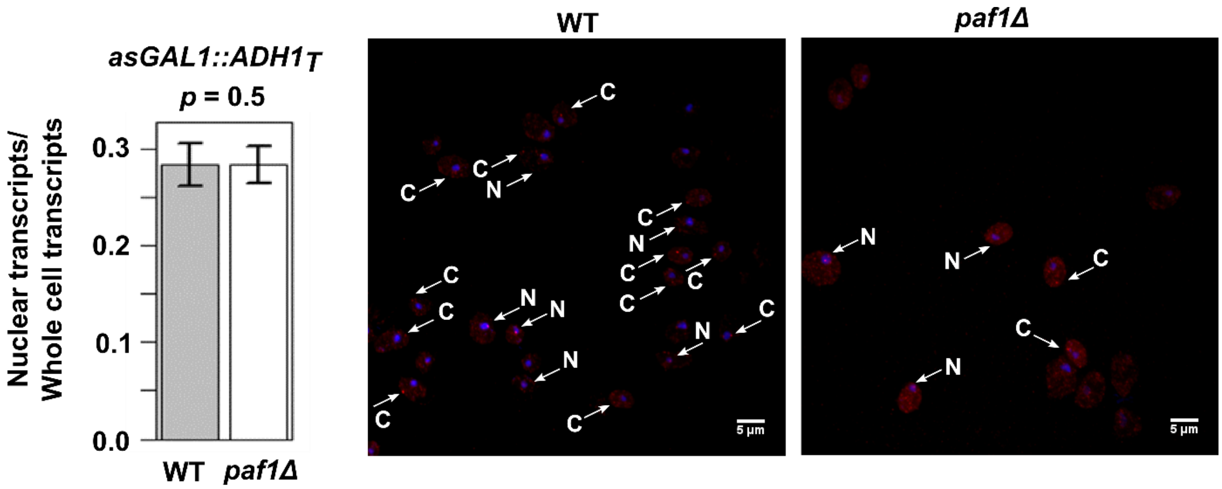

Figure S4

Figure S4, Related to Figure 5. RNA FISH for antisense transcripts at a truncated version of *GAL1* (*GAL1::ADH1*$_T$) in the strains indicated (Murray et al., 2015). Cells were grown in glucose, where the antisense transcript is stably expressed but the galactose inducible *GAL1:ADH1*$_T$ sense transcript is not. Strand-specific probes were used. Bar chart showing the mean ratio of nuclear transcripts/total transcripts. Error bars show the S.E.M., p=0.5, n=3. Representative images showing cells with nuclear (N) or cytoplasmic (C) transcripts used to calculate the ratios in the bar charts.

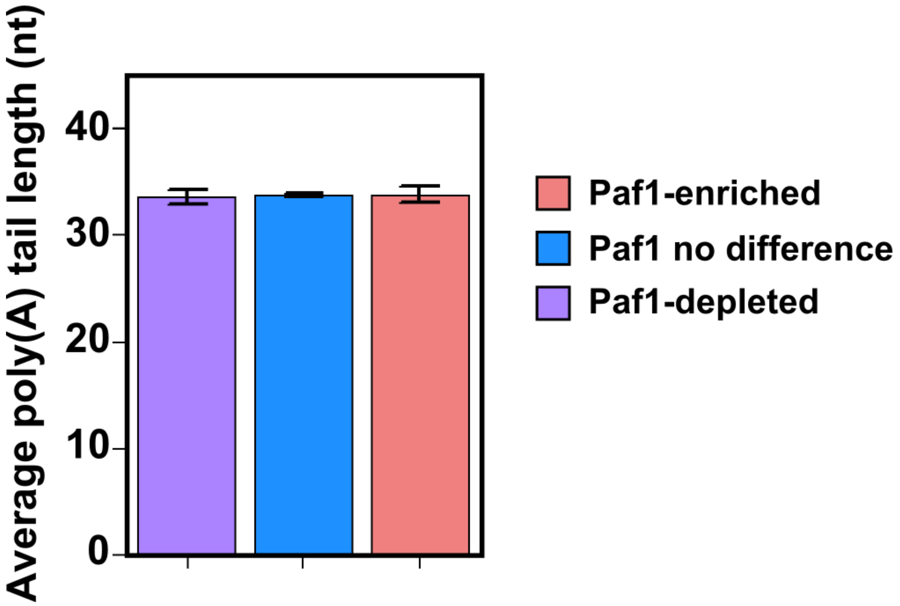

Figure S5, Related to Figures 5 and 6. Differential Paf1 levels do not affect poly(A) tail length. mRNA genes were split into Paf1-enriched (red), Paf1-depleted (purple) and no significant difference in Paf1 (blue) groups ($p < 0.05$). The bar chart shows the mean average poly(A) tail length of transcripts encoded by each group. Error bars show the S.E.M. Transcript poly(A) tail lengths were obtained from Subtelny et al., (2014).

Table S1, Relates to Figure 1-6. Read counts for TEF-seq and NET-seq. Read counts for individual and combined TEF-seq and NET-seq biological replicates before and after subtraction of signal that is not specific to the tagged factor using signal from an untagged control strain (no-tag).

Table S2, Relates to Figures 1-3. Factors subject to TEF-seq.

Table S3, Relates to Figures 4-6. Genes showing enrichment or depletion for Paf1 in yeast cultured in glucose or after 5, 15 and 60 minutes in galactose. Data is shown as log2 FoldChange (log2 relative Paf1 to Rpb3 occupancy ratio). Padj is p value adjusted for multiple testing using the Benjamini-Hochberg method.

Table S4, Relates to Figure 5. PAR-CLIP data analysis. *p* values (Welch's T-test) calculated to test whether there is a significant increase or decrease in the mean average PAR-CLIP factor counts per nucleotide on transcripts encoded by significantly (p value < 0.05) Paf1-enriched or Paf1-depleted mRNA genes, respectively, compared to mRNA genes showing no significant enrichment or depletion for Paf1**.**

Table S5, Relates to Figures 5 and 6. Primers used for qPCR.

**Gene**	**Forward primer sequence**	**Reverse primer sequence**
YPR156C/TPO3	GTGGATCTAAATCCAACATG	ACGGGGGCTTTGGAGCTCAC
YGR138C/TPO2	CTCGTGCAAATCAACTTAAG	GGCGCATTGATATCAGGCAC
YOL016C/CMK2	TGTTCATAGGGATTTGAAAC	CTTCAATTGTTTAGCTATAC
YOL086C/ADH1	GCCACTGACGGTGGTGCTCA	GGCACCAGCTGGCATACCGA

YDR399W/HPT1	GTCGGCAAACGATAAGCAAT	ATGAAACCACCACCACCAAT
YDL052C/SLC1	ATTGACACCTTGAATAAAGG	ACATTGTCAGCTCACTCGTG
YBR005W/RCR1	CAGATGATTATGTTCCTGAG	ATGCAGCCTTATCGTTGGGG
YCR012W/PGK1	GCGTGTCTTCATCAGAGTTG	AGTGAGAAGCCAAGACAACG
YGR033C/TIM21	ACGTCACTGCTTAGAACAAG	TTGAGGCCATAATGGCTTAG
YLR194C/NCW2	CGACATCAACATCAACGGAC	GGCTGAGGACGGTAGCCACC
YLR414C/PUN1	TGACATACTACAACAATTTG	TGATCCAGCGCAATACGTTG
YBR296C/PHO89	TTACTTTAAGTCCACTGATG	ACAGAAACAGTTGTTCCAAG
YGR108W/CLB1	GATGATCCCTTAATGGTGAG	TATGCTTATAGAGATTCGCC
YEL021W/URA3	AGTACTCTGCGGGTGTATAC	TGCCGCCTGCTTCAAACCGC

Table S6, Relates to Figure 5. Probes for RNA FISH.
