# Supplementary material for: Paf1 Has Distinct Roles in Transcription Elongation and Differential Transcript Fate
Source: Mol Cell. 2017 Feb 16;65(4):685–698.e8. doi: 10.1016/j.molcel.2017.01.006 (PMC5316414; doi:10.1016/j.molcel.2017.01.006)
Supplement: Document S2. Article plus Supplemental Information [file mmc7.pdf]

Molecular Cell

Article

Paf1 Has Distinct Roles in Transcription Elongation and Differential Transcript Fate

Graphical Abstract

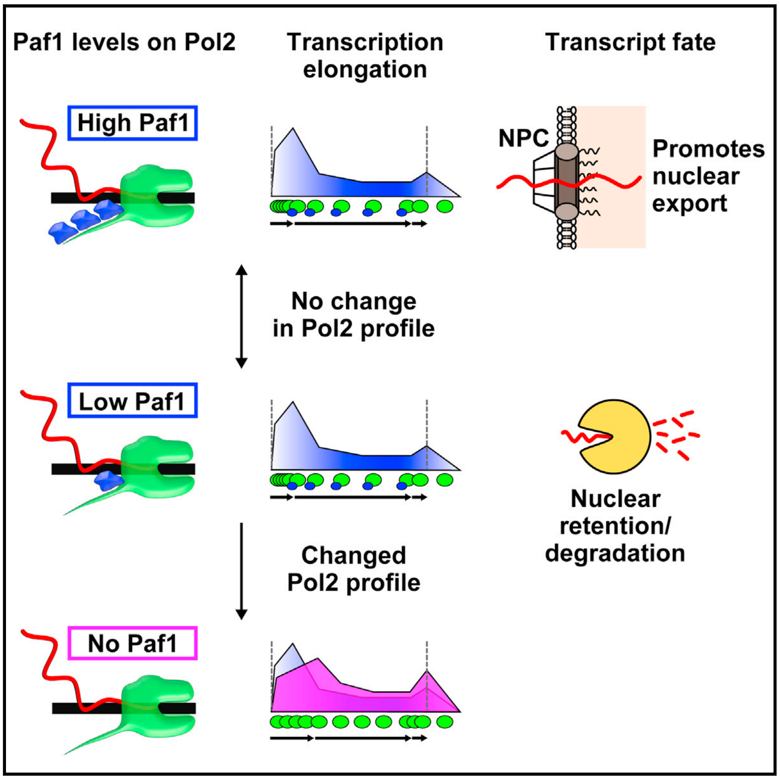

Authors

Harry Fischl, Françoise S. Howe, Andre Furger, Jane Mellor

Correspondence

jane.mellor@bioch.ox.ac.uk

In Brief

Fischl et al. show that different genes are transcribed by RNA polymerase associated with different levels of the elongation factor Paf1. Paf1 levels influence whether transcripts are retained in the nucleus or exported to the cytoplasm, a function distinct from Paf1's role in transcription elongation.

Highlights

- Strand-specific maps of Paf1 on Pol2 using TEF-seq
- Paf1 influences transcription elongation at all genes
- Paf1 is dynamically enriched/depleted on Pol2, independent of its role in elongation
- Paf1 enrichment promotes nuclear export of the encoded transcripts

Fischl et al., 2017, Molecular Cell *65*, 685–698
February 16, 2017 © 2017 The Authors. Published by Elsevier Inc.
http://dx.doi.org/10.1016/j.molcel.2017.01.006

CellPress

Paf1 Has Distinct Roles in Transcription Elongation and Differential Transcript Fate

Harry Fischl,[1] Françoise S. Howe,[1] Andre Furger,[1] and Jane Mellor[1,2,*]
[1]Department of Biochemistry, University of Oxford, South Parks Road, Oxford OX1 3QU, UK
[2]Lead Contact
*Correspondence: jane.mellor@bioch.ox.ac.uk
http://dx.doi.org/10.1016/j.molcel.2017.01.006

SUMMARY

RNA polymerase II (Pol2) movement through chromatin and the co-transcriptional processing and fate of nascent transcripts is coordinated by transcription elongation factors (TEFs) such as polymerase-associated factor 1 (Paf1), but it is not known whether TEFs have gene-specific functions. Using strand-specific nucleotide resolution techniques, we show that levels of Paf1 on Pol2 vary between genes, are controlled dynamically by environmental factors via promoters, and reflect levels of processing and export factors on the encoded transcript. High levels of Paf1 on Pol2 promote transcript nuclear export, whereas low levels reflect nuclear retention. Strains lacking Paf1 show marked elongation defects, although low levels of Paf1 on Pol2 are sufficient for transcription elongation. Our findings support distinct Paf1 functions: a core general function in transcription elongation, satisfied by the lowest Paf1 levels, and a regulatory function in determining differential transcript fate by varying the level of Paf1 on Pol2.

INTRODUCTION

Nuclear export of transcripts via the nuclear pore complex is a key step in eukaryotic gene expression (Wickramasinghe and Laskey, 2015). Although the machinery controlling RNA export is highly conserved, there are many examples of selective RNA export. However, the regulatory processes controlling preferential export of some RNAs over others are not understood. One well studied example of selective RNA export in yeast concerns the generally distinct fate of mRNAs compared with long noncoding RNAs (lncRNAs) (Tuck and Tollervey, 2013). Currently, the distinction between mRNAs and lncRNAs is believed to be made during 3′ end formation of the transcript, following transcription elongation but prior to the acquisition of export competence, with the length of the poly(A) tail being a determinant for this process (Tuck and Tollervey, 2013). The lncRNAs, and some 20% of mRNAs that behave like lncRNAs, are preferentially captured and retained by Mtr4-dependent surveillance. These transcripts are subsequently degraded in the nucleus by the nuclear exosome and are characterized by their reduced association with the conserved nuclear-cytoplasmic export factor Mex67/NXF1/TAP (tandem affinity purification) (Tuck and Tollervey, 2013). Efficient nuclear export of a transcript requires RNA binding proteins such as Nab2, controlling poly(A) tail length, and an adaptor protein such as Yra1/REF, linking the transcript to Mex67 and the nuclear pore complex (Baejen et al., 2014; Hieronymus and Silver, 2003; Tuck and Tollervey, 2013). However, there are likely to be multiple parallel export pathways, with the export of particular mRNAs occurring at different rates in association with particular combinations of poly(A) binding proteins and adaptors. Mex67 links elongation to transcript export as co-transcriptionally deposited monoubiquitylation of lysine 123 on histone H2B (H2Bub) facilitates recruitment of Mex67 to the transcript (Vitaliano-Prunier et al., 2012). The deposition of H2Bub requires the Paf1 complex (Paf1C), containing Paf1 (RNA polymerase II [Pol2]-associated factor 1), in the Pol2 transcription elongation complex (TEC) (Tomson and Arndt, 2013; Yang et al., 2016). Paf1C was originally described as interacting with the TATA binding protein, the transcription elongation factors (TEFs) Spt4-Spt5, and facilitates chromatin transcription (FACT), and, in addition to controlling deposition of H2Bub, Paf1C has been implicated in many aspects of gene regulation (Kowalik et al., 2015; Poli et al., 2016; Tomson and Arndt, 2013; Yang et al., 2016), including release of the promoter-proximal paused Pol2 into productive elongation (Chen et al., 2015; Yu et al., 2015) and transcription elongation on DNA or chromatin templates (Rondón et al., 2004; Tous et al., 2011). Consistent with a direct role in elongation, chromatin immunoprecipitation (ChIP) reveals accumulation of Paf1 over the transcribed region of genes (Mayer et al., 2010). Currently, our understanding of the relationship between TEFs such as Paf1 and elongating Pol2 is limited because ChIP, the technique used to capture TEF interactions in chromatin, cannot resolve the DNA strand involved (Mahony and Pugh, 2015). This problem is compounded by recent observations showing that budding yeast, fly, and mammalian genomes are extensively transcribed, often on both strands of DNA (reviewed in Mellor et al., 2016, and Murray and Mellor, 2016). To address exactly how Paf1 influences transcription elongation and transcript fate, we applied nucleotide resolution strand-specific techniques to map (1) Pol2 on genes in the absence of Paf1 (by native elongating transcript sequencing [NET-seq]) and (2) Paf1 association with elongating Pol2 (by TEF-associated nascent elongating transcript sequencing [TEF-seq], an adaption of NET-seq). Here we show that Paf1 controls the differential nuclear export of

© 2017 The Authors. Published by Elsevier Inc.
This is an open access article under the CC BY license (http://creativecommons.org/licenses/by/4.0/).

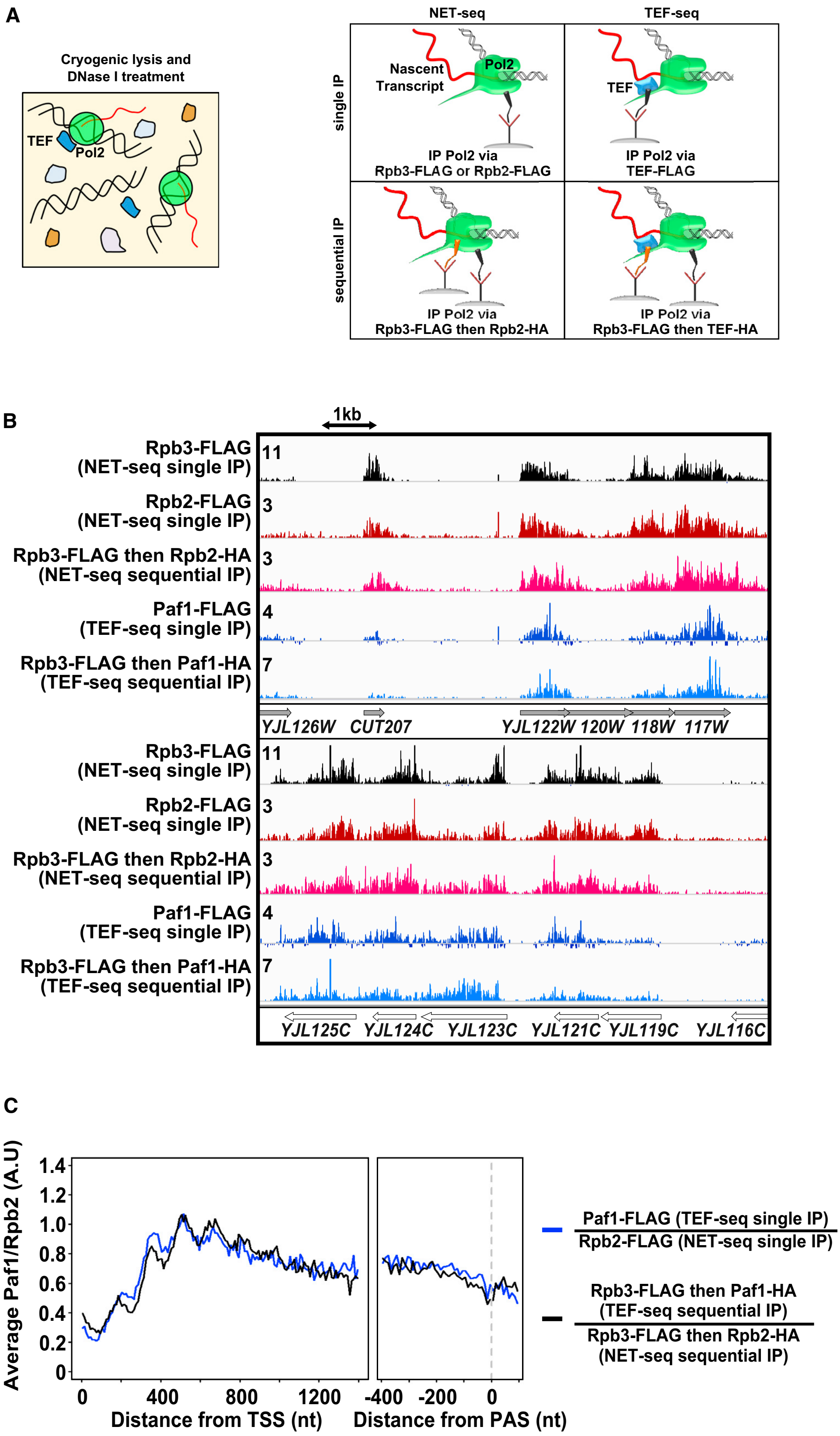

(legend on next page)

mRNAs and preferential nuclear retention of the majority of lncRNAs and some mRNAs. Paf1 shows selective enrichment on, or depletion from, Pol2-transcribing RNAs that are also selectively enriched or depleted for 3′ end processing and export factors. Paf1 enrichment/depletion on Pol2 is dynamic and controlled by environmental factors via promoters, suggesting that the fate of transcripts is equally dynamic and determined before their transcription. Paf1 also functions to control Pol2 distribution on genes during elongation, but this function is common to all genes and is distinct from its role in orchestrating differential nuclear export of specific transcripts. Together, our findings support distinct functions for Paf1C associated with Pol2, a core function in Pol2 elongation at all genes and a regulatory function determining differential transcript fate at selected genes.

RESULTS

Mapping Strand-Specific Paf1 Association with Pol2 Using TEF-Seq

To map the position of Paf1 on elongating Pol2, we immunoprecipitated) FLAG-tagged Paf1 and sequenced the nascent RNA, purified from the active site of co-immunoprecipitated Pol2, from the 3′ end (Figure 1A; Tables S1 and S2; Figures S1 and S2). This technique, called TEF-seq, is related to NET-seq, which maps the position of the 3′ nucleotide of the nascent RNA in transcriptionally engaged Pol2 (Churchman and Weissman, 2011). Alignment of reads shows the position of the final incorporated nucleotide, giving a strand-specific nucleotide resolution map of transcriptionally engaged Pol2 associated with Paf1. Replicates show that TEF-seq data are reproducible (Figure S1H). To ensure that our map (Figure 1B) reports only the signal from Pol2 associated with Paf1, we used two important controls. First, we removed the background signal that is not specific to tagged Paf1 using material present in an immunoprecipitation (IP) from a strain lacking a FLAG tag (no-tag control; STAR Methods; Figures S1E–S1G). Second, to show that the RNA recovered is specifically associated with Pol2, we subjected Paf1 to single or sequential IP procedures (Paf1-FLAG or Rpb3-FLAG and then Paf1-HA) and normalized to levels of Pol2 also subjected to single or sequential IPs (Rpb2-FLAG or Rpb3-FLAG and then Rpb2-HA) by tagging the Rpb3 and/or Rpb2 components of Pol2 (Figure 1; STAR Methods). The Pol2-normalized Paf1 metagene profiles for the single and sequential IPs are similar, suggesting that TEF-seq captures only Paf1 associated with elongating Pol2 and not from a Pol2-independent but direct association of the Paf1C with RNA. Thus, we are capturing Paf1 specifically associated with Pol2, although Paf1 could be associated with Pol2 via the transcript emanating from its active site (Dermody and Buratowski, 2010), via the phosphorylated C-terminal domain (CTD) of its largest subunit, Rpb1 (Custódio and Carmo-Fonseca, 2016; Qiu et al., 2012) or via another TEF (Tomson and Arndt, 2013).

Paf1 Oscillates on Pol2 during Transcription through Chromatin

The association of Paf1 with elongating Pol2 is not uniform but shows a remarkable periodicity (~170 nt), reminiscent of phased positioned nucleosomes (Jiang and Pugh, 2009; Figures 1C and 2A). To examine this in more detail, TEF-seq profiles were obtained for the Pol2-associated histone chaperones Spt6 and Spt16 (Tables S1 and S2; Figures S1 and S2). The Rpb3-normalized metagene profiles for Spt6, Spt16, and Paf1 revealed their dynamic behavior during transcription through chromatin (Figure 2). Relative to the center point of nucleosomes +2 to +5, these factors oscillate around nucleosomes, with peaks and troughs separated by ~80 nt, offset for Spt6 compared with Spt16 and Paf1, which are known to interact (Krogan et al., 2004; Squazzo et al., 2002). The marked phasing offset between Spt6 and Spt16 oscillations may reflect their preferential affinities for H3 or H2A-H2B dimers, respectively (Bortvin and Winston, 1996; Hondele et al., 2013). The ability to detect the dynamics of transcription through chromatin reflects the high resolution of the TEF-seq data.

Paf1 Is Enriched and Depleted on Pol2

The high resolution of these data gives us the opportunity to study Paf1 levels on Pol2 transcribing different genes. TEF-seq reads for Paf1 from the transcript start site (TSS) + 400 nt to the transcript end site (TES) − 200 nt were counted, thus excluding regions in which Paf1 shows large changes in its association with Pol2. Differential occupancy was assessed by comparing the Paf1-specific counts to counts for Rpb3 across the same regions of mRNA genes. Paf1 shows a large range in relative occupancy levels, with 138 genes significantly ($p < 0.05$) Paf1-enriched and 153 Paf1-depleted (Figures 3A and 3B; Table S3; see STAR Methods for selection of gene groups for statistical analysis). Paf1 is also depleted from Pol2 on lncRNA genes regardless of

Figure 1. Mapping Paf1 on Pol2 Using TEF-Seq

(A) Schematic showing single or sequential immunoprecipitation of Pol2 (via the components Rpb3 or Rpb2, NET-seq) or TEF:Pol2 complexes (TEF-seq) via epitope tags from cryogenically lysed and DNase I-treated yeast extracts.

(B) Examples of Rpb3 and Rpb2 NET-seq and Paf1 TEF-seq profiles on individual genes. Images in integrated genome viewer (IGV) (Thorvaldsdóttir et al., 2013) show loci on the Watson (top, filled arrows) or Crick (bottom, open arrows) strands. Scales allow relative changes to be visualized and are proportional to each other across all IGV images.

(C) Comparison of Rpb2-normalized Paf1 metagene profiles from single (blue) or sequential (black) IP procedures aligned at the TSS or PAS (dashed line, right). Pol2-normalized (using either Rpb2 or Rpb3 NET-seq data) TEF metagene profiles are calculated as follows. TEF/Pol2 (TEF-seq counts/NET-seq counts) ratios are calculated for each 10-nt bin across the specified region. For TSS-aligned plots, the final 200 nt of each gene before the TES is excluded. For PAS or TES-aligned plots, the initial 500 nt after the TSS of each gene is excluded. These ratios are multiplied by a TEF and Pol2 subunit-specific scaling factor to enable visualization on the same arbitrary linear scale. The mean (scaling factor × TEF)/Pol2 ratios are then calculated for each 10-nt bin across all genes after first excluding the top 10% and bottom 10% of ratios to prevent single-nucleotide spikes from skewing the mean (see STAR Methods for details).

See also Tables S1 and S2 and Figures S1 and S2.

A

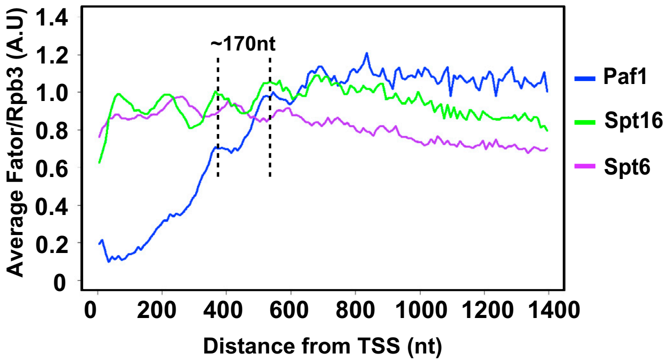

B

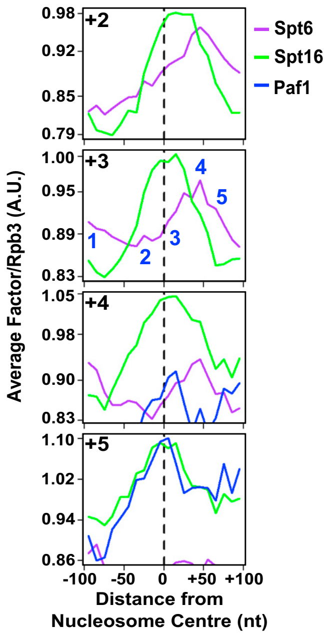

C

	+2	+3	+4	+5
Spt6	8.7×10^{-19}	2.3×10^{-14}	1.0×10^{-12}	0.89
Spt16	2.3×10^{-9}	1.6×10^{-16}	1.1×10^{-4}	2.9×10^{-7}
Paf1	0.997	6.5×10^{-2}	8.9×10^{-4}	3.6×10^{-5}

D

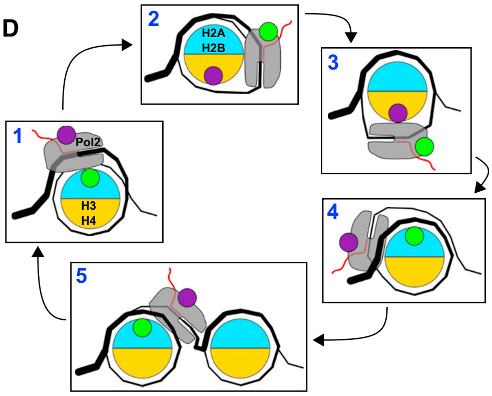

Figure 2. Levels of Paf1 Oscillate on Pol2

(A) Rpb3-normalized TEF metagene profiles for Paf1, Spt6, and Spt16 from the TSS to +1400 nt averaged across mRNA genes (see Figure 1C for details).

(B) Rpb3-normalized TEF metagene profiles aligned to the center (dashed line) of nucleosomes +2 to +5 (relative to the TSS) averaged across mRNA genes.

(C) p Values (one-tailed Student's t test) calculated to test whether there is a significant difference in the mean TEF/Rpb3 ratio at the center compared with the center +50 nt of each nucleosome.

(D) Schematic showing five stages of the oscillations between Pol2, Spt6, Spt16, Paf1, and nucleosomes (the green circle is Paf1 and Spt16, and purple is Spt6). The equivalent positions (1–5) are shown in (B).

See also Tables S1 and S2 and Figures S1 and S2.

whether they encode stable or unstable transcripts (Figures 3C and 3D). No significant difference in transcript levels between the Paf1-FLAG- and Rpb3-FLAG-tagged strains was evident for Paf1-enriched or -depleted genes (Figure S3), showing that the Paf1 and Rpb3 epitope tags are not responsible for the differential levels observed. Finally, enrichment and depletion of Paf1 is also observed at the same sets of genes when the read counts for Paf1 in the single or sequential IPs are normalized to read counts for Rpb2 (instead of Rpb3) in single or sequential IPs, respectively (Figures 3B and 3D).

Genes with Paf1-Enriched Pol2 Share Regulatory Features

Paf1-enriched mRNA genes share distinctive features not seen in Paf1-depleted mRNA genes. First, there is a significant enrichment ($p < 0.00023$) for divergently expressed gene pairs, 100 pairs within the 1,200 most Paf1-enriched genes, selected by ordering the genes by their log_2 relative Paf1-to-Rpb3 ratio. This suggests that the shared bi-directional promoter between these divergent pairs, which are often, but not always, co-regulated (Yan et al., 2015), may be responsible for promoting Paf1 enrichment on both genes. This enrichment for divergent pairs is also present within the original class of 138 significantly ($p < 0.05$) Paf1-enriched genes (four pairs, $p < 0.017$). There is no significant enrichment for divergent pairs within the 1,200 most Paf1-depleted genes (70 pairs, $p = 0.73$). Second, Paf1-enriched genes share regulatory features (Figure 3E; Figure S3). They are regulated by transcription factors such as Sfp1, Haa1, and Cbf1/Cpf1 (a MYC homolog) (Hu et al., 2007; Keller et al., 2001; Mellor et al., 1990). Interestingly, degradation of promoter-bound MYC releases PAF1C for transfer onto Pol2 during elongation in mammalian cells (Jaenicke et al., 2016). In addition, the promoters of significantly ($p < 0.1$) Paf1-enriched genes are enriched with DNA sequence elements within 500 nt of the TSS, such as AAAATTTT/C (104 of 229, $p = 1.9 \times 10^{-9}$) and CTCATCG/T (81 of 229, $p = 6.4 \times 10^{-9}$), binding sites for Sfp1 and Dot6/Tod6, respectively, often present together in promoters (Jorgensen et al., 2002). This supports a role for the promoter in determining Paf1 enrichment on Pol2. To validate this, we replaced the coding regions of Paf1-enriched *CMK2* and Paf1-depleted *SLC1* with *URA3* and immunoprecipitated the transcripts associated with FLAG-tagged Rpb3 or FLAG-tagged Paf1. Assessment of levels of nascent transcripts immunoprecipitated from the hybrid genes revealed that the Pol2 transcribing *URA3* from the promoter of the Paf1-enriched gene is more Paf1-enriched than when transcribing *URA3* from the promoter of the Paf1-depleted gene (Figure 3F). This further implicates the promoter in determining levels of Paf1 on Pol2. Next we asked how the promoter influences enrichment or depletion of Paf1 on Pol2 transcribing different genes.

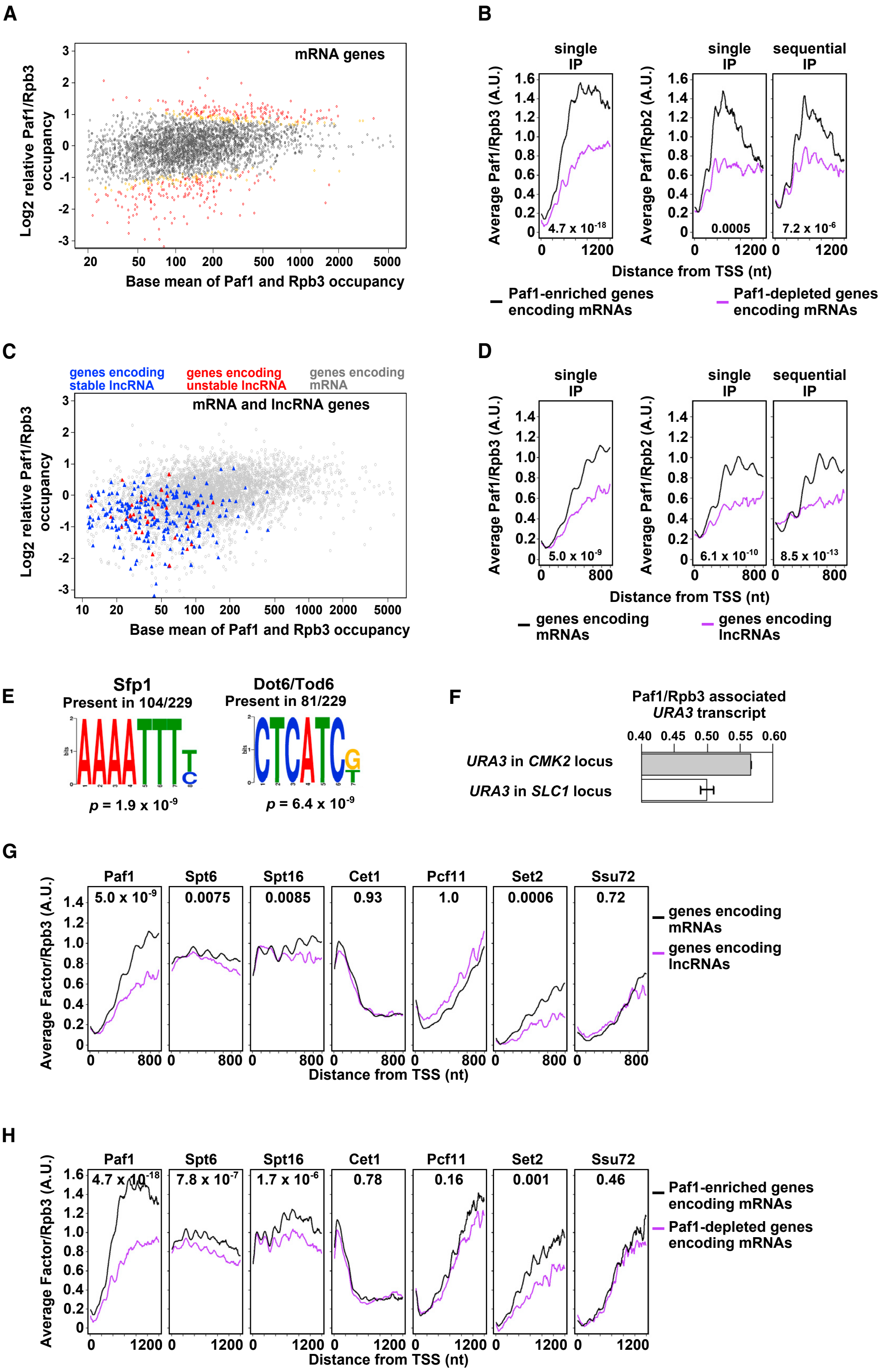

(legend on next page)

Differential Levels of Paf1 on Pol2 Reflect Levels of Serine 2 and 5 Phosphorylation of the Pol2 CTD

One feature of lncRNA genes, transcribed by Paf1-depleted Pol2, is that Pol2 shows reduced levels of Ser2-phosphorylated CTD on Rpb1 (Milligan et al., 2016; Murray et al., 2015). This is consistent with the documented role for Paf1C in determining levels of Ser2 phosphorylation on the Rpb1 CTD in Pol2 (Dronamraju and Strahl, 2014). Because Paf1C can also interact with Pol2 via its Ser2-phosphorylated CTD (Qiu et al., 2012), this positive feedback provides a possible explanation for how differential recruitment, controlled by the promoter, is maintained during transcription. To relate enrichment or depletion from Pol2 to the phosphorylation status of the CTD, we used TEF-seq to assess levels of six additional TEFs on Pol2 (Tables S1 and S2). These include (1) Set2, the lysine 36 on histone H3 (H3K36) methyltransferase, and Pcf11, required for 3′ end formation, both of which, like Paf1, show some dependence on Ser2-phosphorylated CTD for their association with Pol2; (2) Spt6 and Spt16, which are known to interact with Paf1; (3) Cet1, a capping enzyme that interacts with Ser5-phosphorylated CTD; and (4) Ssu72, a Ser5 phosphatase (Figures 3G and 3H; Table S2; Figure S2). Set2, Spt6, and Spt16 show significantly decreased recruitment to Pol2 on lncRNA genes compared with mRNA genes and on the 1,000 most Paf1-depleted genes compared with the 1,000 most Paf1-enriched genes, whereas no significant decrease is observed for Pcf11, Cet1, or Ssu72. Because Pcf11 and Cet1 interact with CTD repeats phosphorylated at Ser2 or Ser5 alone (Table S2), respectively, differential levels of these modifications are unlikely to be related to levels of Paf1. By contrast, Set2 and Paf1 preferentially interact with the CTD when both Ser2 and Ser5 are phosphorylated within the same and/or adjacent repeats (Kizer et al., 2005; Qiu et al., 2012). This analysis suggests that differential Ser2 phosphorylation, only within or next to Ser5 phosphorylated repeats, is the key to maintaining differential Paf1 during transcription. Finally, because a *paf1Δ* strain lacking all Paf1 protein shows reduced levels of both Ser2 phosphorylation and Pcf11 on genes (Tomson and Arndt, 2013) but Pcf11 does not show significantly decreased levels on Paf1-depleted genes (Figures 3G and 3H), we propose that a low level of Paf1, equal to that on the most Paf1-depleted genes identified by our TEF-seq analysis, may determine a level of CTD repeats phosphorylated at Ser2 alone sufficient for Pcf11 recruitment.

Paf1-Enriched or Depleted Pol2 on Genes Is Not Related to Transcription Elongation

To address whether Paf1 enrichment/depletion on Pol2 is related to the documented role of Paf1 in transcription elongation (Rondón et al., 2004; Tous et al., 2011), we subjected a *paf1Δ* strain lacking all Paf1 protein to NET-seq, which effectively captures all forms of engaged Pol2 on the genome (Churchman and Weissman, 2011; Nguyen et al., 2014). Pol2 metagene profiles (Figure 4A), heatmaps showing Pol2 profiles on all genes (Figure 4B), and Pol2 profiles on individual genes (Figure 4C) reveal distinct differences. The most notable differences in the *paf1Δ* strain are the shifts in the position of the 5′ peak toward the 3′ end and increased reads over the 3′ region of genes relative to reads over the 5′ region, with a notable peak just preceding the polyadenylation site (PAS). To see whether genes with Pol2 enriched or depleted for Paf1 have different distributions of Pol2 over their transcribed regions, we compared the metagene profiles for each class (Figure 4D). The genes with Paf1-enriched or Paf1-depleted Pol2 showed an identical profile after controlling for gene length. Importantly, both profiles were different from that obtained from a *paf1Δ* strain lacking all Paf1 protein. Thus, a certain level of Paf1 ensures a normal profile of Pol2 during transcription elongation on all genes, which must be equivalent to or below that on the most Paf1-depleted gene identified by our TEF-seq analysis. If Paf1 enrichment or depletion is not related to elongation, then what is it doing?

Paf1-Enriched Pol2 Encodes Transcripts Enriched for RNA Binding Proteins Involved in Processing and Nuclear Export

We took a bioinformatics approach to learn more about the consequences of Paf1 enrichment or depletion on Pol2. Analysis of photoactivatable ribonucleoside-enhanced crosslinking and immunoprecipitation (PAR-CLIP) datasets in which RNA-bound factors are directly UV cross-linked to transcripts revealed that Paf1-enriched or -depleted genes encode transcripts that are similarly enriched or depleted for RNA processing and nuclear export factors. Mean PAR-CLIP counts per nucleotide for 23 RNA-associated factors already normalized for transcript

Figure 3. Differential Levels of Paf1 on Pol2 Are Determined by the Promoter and CTD Phosphorylation Status

(A and B) Differential Paf1 occupancy at mRNA genes.

(A) Scatterplot showing the base mean occupancy against the $\log_2$ relative Paf1-to-Rpb3 occupancy ratio for each gene. Yellow points, $0.05 \leq p < 0.1$. Red points, $p < 0.05$.

(C and D) Differential Paf1 occupancy at lncRNA (triangles) and mRNA (circles) genes.

(C) Scatterplot showing the base mean occupancy against the $\log_2$ relative Paf1-to-Rpb3 occupancy ratio for each gene.

(E) Sequence motifs enriched within 500 nt upstream of the most significantly ($p < 0.1$) Paf1-enriched genes. Discriminative Regular Expression Motif Elicitation (DREME) (Bailey, 2011) was used to uncover and display the motifs.

(F) Bar chart showing Paf1 enrichment on Pol2 (Rpb3) for the hybrid constructs indicated. The *URA3* coding region replaces that of *CMK2* or *SLC1* (the endogenous genes are Paf1-enriched and Paf1-depleted, respectively). Error bars show the SEM (n = 2).

(B, D, G, and H) Pol2-normalized TEF metagene profiles from the TSS to +800 nt (D and G) or +1,200 nt (B and H) averaged across all mRNA genes (black) and all lncRNA genes (purple, D and G) or the 1,000 most Paf1-enriched or -depleted mRNA genes (B and H), selected by ordering the genes by their $\log_2$ relative Paf1-to-Rpb3 occupancy ratio (see Figure 1C for details). Profiles are smoothed by calculating a running mean average using a moving window containing five 10-nt bins. p Values (one-tailed Welch's t test) are calculated to test whether there is a significant difference in the ratios of the two groups at each 10-nt bin from 400–700 nt (D and G) or 400–1,100 nt (B and H). The upper quartile of these is taken, meaning that 75% of p values are lower than that shown.

See also Table S3.

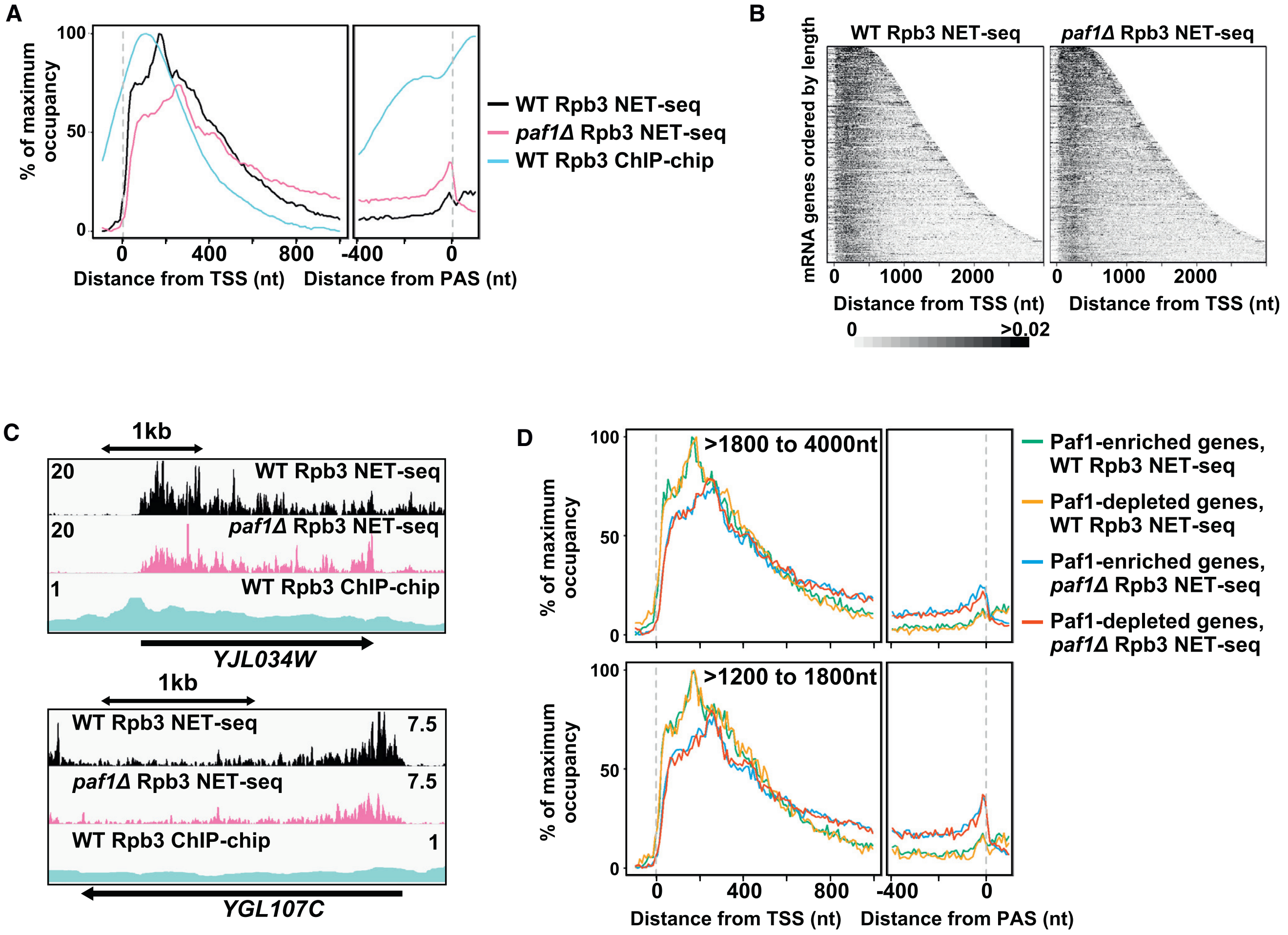

Figure 4. Distinct Functions for Paf1 in Elongation and RNA Fate

(A) Metagene occupancy profiles averaged across mRNA genes using Rpb3 chromatin immunoprecipitation on a microarray chip (ChIP-chip) data (Mayer et al., 2010) and Rpb3 NET-seq data for WT and *paf1*Δ strains. ChIP-chip and WT NET-seq profiles are plotted on a linear scale from minimum to maximum, and the *paf1*Δ profile is plotted so that the area under the curve (AUC) between the TSS and the PAS is the same as the WT AUC (see STAR Methods for details).

(B) Heatmaps showing Pol2 occupancy determined by Rpb3 NET-seq over all genes ordered by length (see STAR Methods for details).

(C) Images in IGV from selected loci (see Figure 1 for details).

(D) Metagene NET-seq occupancy profiles averaged across mRNA genes restricted by length (>1,800–4,000 nt or >1,200–1,800 nt) that are either Paf1-enriched ($\log_2$ relative Paf1-to-Rpb3 occupancy ratio > 0.2) (n = 378 and 449, respectively) or depleted ($\log_2$ relative Paf1-to-Rpb3 occupancy ratio < −0.2) (n = 614 and 335, respectively). Profiles are shown for WT and *paf1*Δ strains with data plotted so that the AUCs between the TSS and the PAS are the same.

See also Tables S1 and S3.

levels were calculated for all transcripts encoded by each Paf1 group: enriched, depleted, or showing no significant enrichment or depletion ($p < 0.05$) (Figure 5A; Table S4; Baejen et al., 2014). Paf1-enriched or depleted genes encode transcripts that are significantly ($p < 0.001$) enriched or depleted for six of the 23 RNA-associated factors (Cft2 [CPSF-100], Mex67 [TAP], Mpe1, Nab2 [ZC3H14], Pab1 [PABPC1], and Npl3) compared with those with no particular Paf1 enrichment or depletion. These factors have a range of functions, including transcript cleavage and polyadenylation (Cft2 and Mpe1), poly(A) binding (Nab2 and Pab1), and nuclear export of transcripts (Mex67 and Npl3). Yra1, an RNA adaptor that interacts with Mex67, Hpr1, and Sub2, components of mRNA export complexes, is also enriched on transcripts encoded by Paf1-enriched genes. Interestingly, Paf1C interacts with Hpr1, Yra1, Npl3, and Nab2 (Table S4).

We used a second dataset that clustered genes into ten groups (I–X) based on the association of their encoded transcripts with RNA binding proteins using crosslinking and cDNA analysis (CRAC), a technique related to PAR-CLIP (Figure 5B; Tuck and Tollervey, 2013). Paf1-enriched genes are enriched in clusters VIII–X. Because the encoded transcripts of the genes in clusters VIII–X are associated with RNA binding proteins involved in transcript export (Mex67) or cytoplasmic RNA processing functions (Xrn1 and Ski2), this confirms the link between Paf1-enriched genes and the preferential export of their encoded transcripts, and clusters with no clear enrichment for

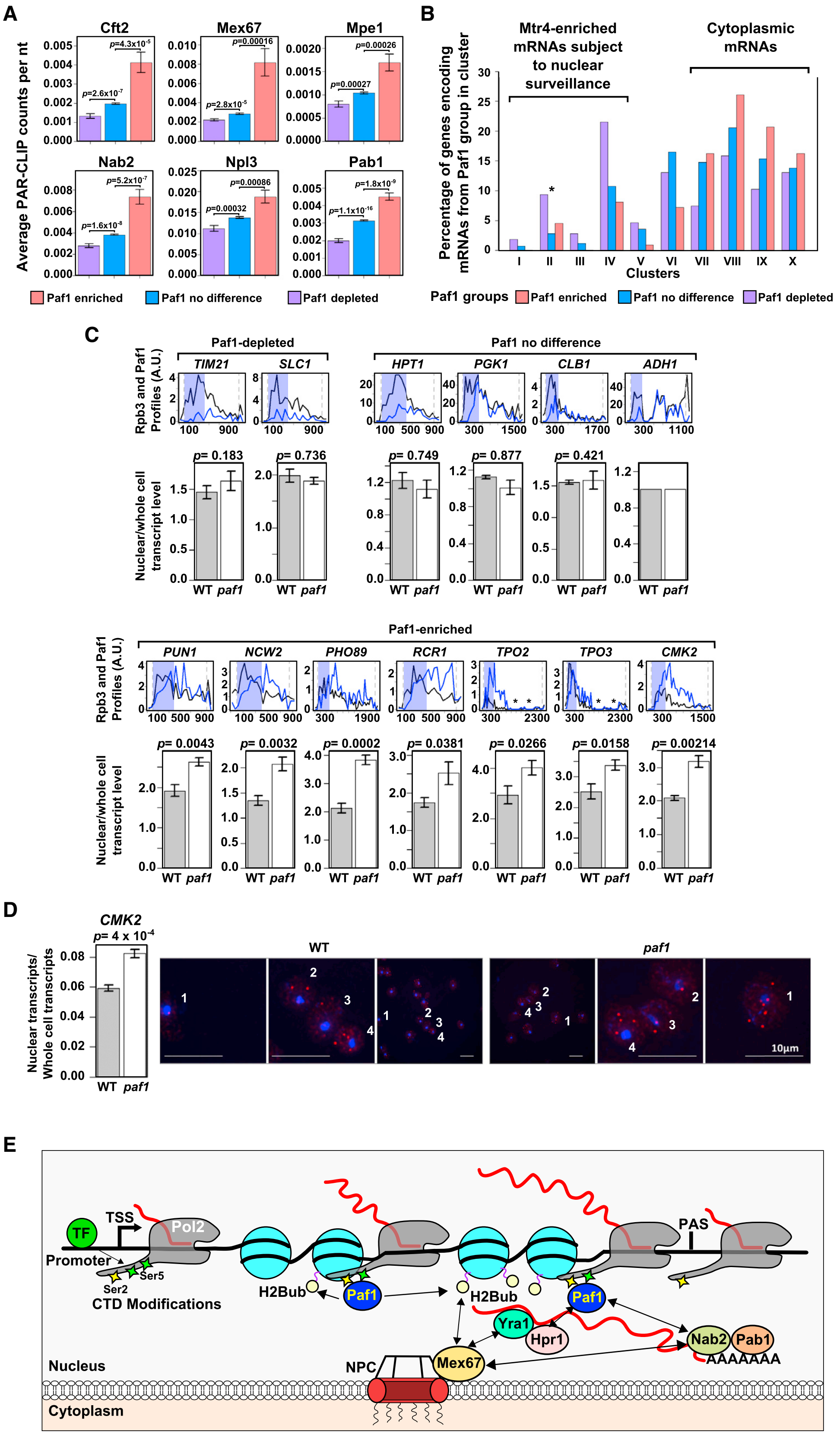

(legend on next page)

Paf1-enriched or -depleted genes (clusters V–VII) illustrate the selective nature of Paf1 function. Importantly, this dataset also contains nuclear surveillance factors (clusters I–IV) such as Mtr4 (SKIV2L2/hMtr4) that target transcripts for processing or degradation by the nuclear exosome. Clusters I–IV, containing Mtr4-associated stable and unstable lncRNAs and 852 of 3,966 mRNAs that are preferentially retained and degraded in the nucleus, are also enriched for transcripts from Paf1-depleted genes. Because the Paf1 groups only contain genes annotated as encoding mRNAs, this association is likely to reflect the mRNAs that are treated similarly to lncRNAs and retained in the nucleus because of low levels of Paf1 on Pol2. This points to a novel role for Paf1 in co-transcriptionally determining selective transcript fate by orchestrating the recruitment of particular RNA binding proteins.

Paf1-Enriched Pol2 Promotes Nuclear Export of the Encoded Transcript

If the processing and export of these selected transcripts depend on levels of Paf1 on Pol2 transcribing the gene, we predict that Paf1-enriched genes would show increased nuclear retention of their encoded transcripts when *PAF1* is ablated (*paf1Δ*). Indeed, this is what we observed by cell fractionation followed by qRT-PCR at transcripts from Paf1-enriched genes (Figure 5C). No significant increase in nuclear retention was observed for transcripts from Paf1-depleted genes or genes at which Paf1 is neither enriched nor depleted. We used single-molecule RNA fluorescence in situ hybridization (FISH) to detect the transcripts from Paf1-enriched *CMK2* in wild-type (WT) and *paf1Δ* backgrounds to confirm these observations (Figure 5D). Counting the number of *CMK2* transcripts in the nucleus and cytoplasm of more than 1,000 individual cells reveals a significant increase in nuclear relative to cytoplasmic transcripts in the absence of Paf1. As expected, an lncRNA showed no change in subcellular localization in the *paf1Δ* background (Figure S4). These data support a link between Paf1-enriched Pol2 on genes and nuclear export of the encoded transcripts (Figure 5E). However, because deletion of Paf1 may result in changes that indirectly affect transcript export, we sought to confirm the link between differential Paf1 enrichment and RNA export using physiological conditions to alter Paf1 levels on Pol2.

Paf1 Enrichment or Depletion on Pol2 Is Dynamic and Related to Differential RNA Export from the Nucleus

Because promoters are involved in Paf1 enrichment on Pol2, we expect enrichment/depletion of Paf1 on Pol2 to be dynamic and change with environmental conditions. We used a simple carbon source shift, transferring cells from glucose- to galactose-containing medium for 5, 15, or 60 min, and compared Paf1 TEF-seq profiles and Rpb3 NET-seq profiles at the three time points with the same data for the cells grown in glucose. We observed significant enrichment or depletion for Paf1 on Pol2 for each sample (Figure 6A; Table S3). Importantly, by comparing how genes that are Paf1-enriched or -depleted under one condition change under the other three conditions, we demonstrate unequivocally that levels of Paf1 on Pol2 are dynamic and that a gene transcribed by Paf1-depleted Pol2 under one condition can be transcribed by Paf1-enriched Pol2 under another condition, and vice versa, independent of levels of transcription. Consistent with a role for transcription factors in differential recruitment of Paf1, 19 of 30 genes (63%) with significantly ($p < 0.05$) Paf1-enriched Pol2 in both glucose and after 60 min in galactose are regulated by Sfp1.

To relate differential Paf1 enrichment to nuclear export of the encoded transcripts, we identified genes that are Paf1-enriched in glucose that also show a large reduction in the levels of Paf1 on Pol2 after 60 min in galactose without any major change in levels of Pol2 under the two conditions (Figure 6B). We predicted

Figure 5. Paf1 Influences the Nuclear Export of Transcripts

(A) Transcripts from Paf1-enriched or Paf1-depleted mRNA genes show increased or decreased binding, respectively, to Cft2, Mex67, Mpe1, Nab2, Npl3, and Pab1. mRNA genes were split into Paf1-enriched (red), Paf1-depleted (purple), and no significant difference in Paf1 (blue) groups ($p < 0.05$). Bar charts show the average PAR-CLIP counts per nucleotide, normalized for differences in transcript levels, for each group for each protein using data from Baejen et al. (2014). Error bars show the SEM p values (Welch's t test) calculated to test the significance of the increase or decrease in the mean of the enriched or depleted group relative to the no significant difference group, respectively.

(B) Transcripts from Paf1-enriched or Paf1-depleted mRNA genes show increased binding to cytoplasmic factors or the nuclear surveillance factor Mtr4, respectively. Bar charts show the percent of mRNA transcripts from each Paf1 group in each of ten clusters defined by Tuck and Tollervey (2013). Transcripts have been clustered according to their association with particular combinations of RNA binding proteins. The asterisk marks cluster II, in which transcripts are associated specifically with the poly(A) binding protein Pab1 but retained in the nucleus for degradation.

(C) Top: profiles showing average relative occupancy for Paf1 (blue) and Rpb3 (black) in 50-nt bins from TSS −100 nt to PAS +100 nt on selected mRNA genes. TSS and PAS are shown by dashed lines. The asterisk marks gaps in the profiles resulting from discarding non-uniquely aligned reads. Blue shading indicates the first 400 nt of each gene, reflecting regions were Paf1 has not reached maximum levels on Pol2. Paf1 levels are multiplied by a scaling factor (2.2) across all profiles to enable visualization of relative changes. Bottom: bar charts showing the mean ratio of nuclear to whole-cell transcript levels for the loci shown in the WT and *paf1Δ* strains, measured using qRT-PCR for transcripts encoded by the genes shown above. Transcript levels are normalized to the level of the *ADH1* transcript in each sample. Error bars show the SEM p values (Welch's t test) calculated to test the significance of the increase in the mean ratio in the *paf1Δ* strain (n = 4).

(D) RNA FISH for *CMK2* transcripts in WT and *paf1Δ* strains. The bar chart shows the mean ratio of nuclear to whole-cell transcripts in more than 200 cells per repeat experiment. Error bars show the SEM p value (Welch's t test) calculated to test the significance of the increase in the mean ratio in the *paf1Δ* strain (n = 4). Also shown are images showing the distribution of transcripts in a wide field and selected images of individual cells for WT and *paf1Δ* strains. Selected individual cells are numbered 1–4. Blue is DAPI, red dots are *CMK2* transcripts (see also Figures S4 and S5).

(E) Schematic showing the known relationships between Paf1 in Paf1C, H2Bub, and RNA processing and export factors (arrows indicate known direct or indirect interactions). Also shown are the features determined in this work, including the role of transcription factors (TFs) and the phosphorylated CTD (Ser2 and Ser5) in selective Paf1 enrichment on Pol2.

See also Tables S4, S5, and S6.

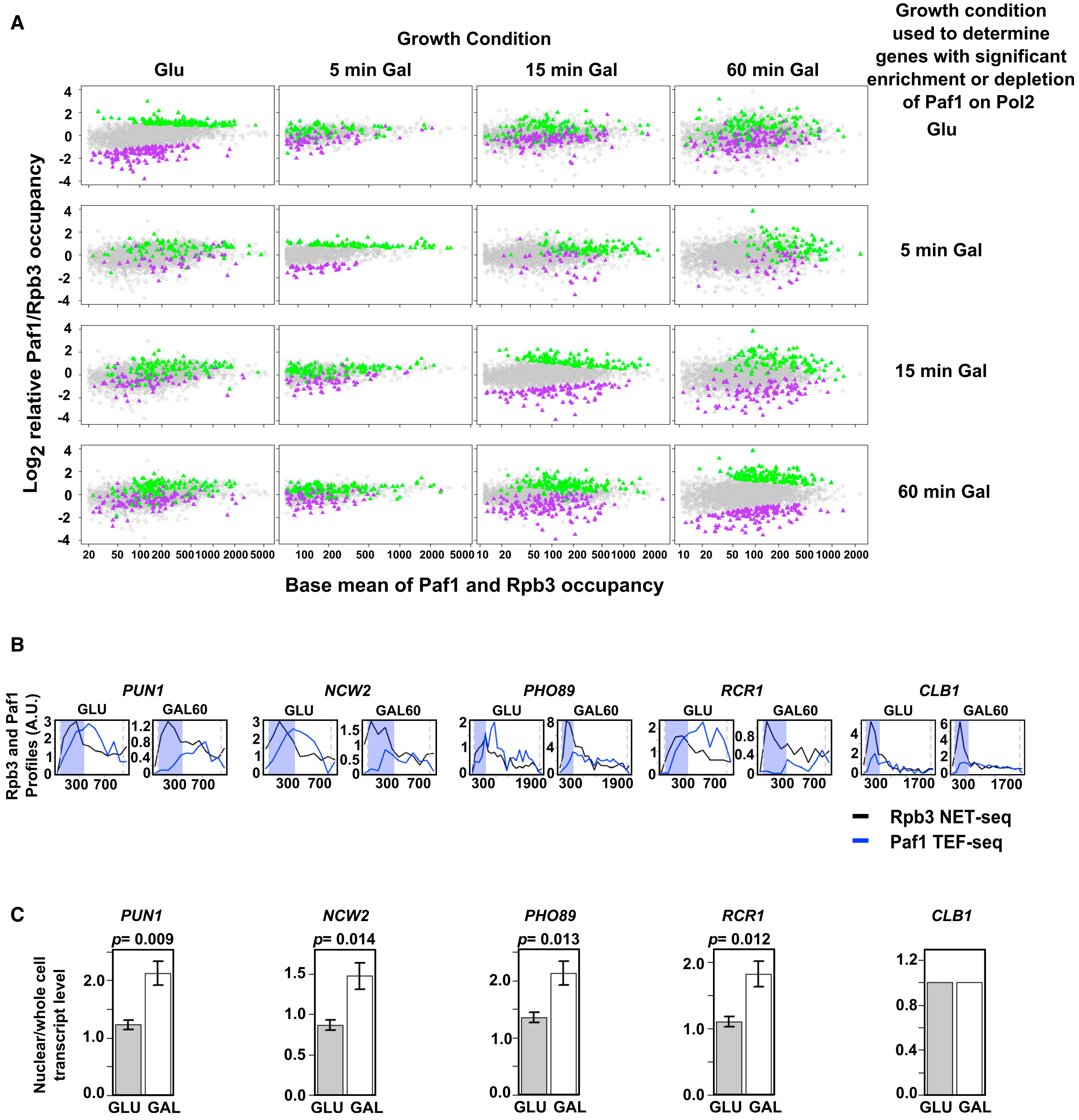

Figure 6. Paf1 Enrichment or Depletion on Pol2 Is Dynamic and Related to RNA Export

(A) Differential Paf1 occupancy at mRNA genes cultured in glucose (GLU) or after 5, 15, or 60 min in galactose (GAL) (columns). The rows show genes that are significantly ($p < 0.05$) enriched (green) or depleted (purple) under each of the four conditions and how the patterns of enrichment or depletion for those genes under one condition changes as the environment changes (growth conditions). For each condition, Paf1 TEF-seq and Rpb3 NET-seq data were obtained in duplicate. Each scatterplot shows the base mean occupancy against the $\log_2$ relative Paf1-to-Rpb3 occupancy ratio for each mRNA gene.

(B) Profiles showing average relative occupancy for Paf1 (blue) and Rpb3 (black) in 100-nt bins across selected mRNA genes. Paf1 levels are multiplied by 2.2 across all GLU profiles and 1.8 across all GAL 60-min profiles because this brings the level of Paf1 compared with Rpb3 to the same level for both conditions for the control gene, *CLB1*, which shows almost no change in relative Paf1-to-Rpb3 occupancy between these two conditions. Pol2 on the selected genes is significantly ($p < 0.05$) Paf1-enriched in glucose, and all show a decrease in the $\log_2$ relative Paf1-to-Rpb3 occupancy ratio greater than 1.2 upon the switch to galactose for 60 min.

(legend continued on next page)

that, in galactose medium, the encoded transcripts should show increased nuclear retention, and, indeed, this is what we observed (Figure 6C). Taken together, these data suggest that the enrichment/depletion of Pol2 with Paf1 during elongation plays a role in determining the selective fate of the nascent transcripts independent of transcription elongation.

DISCUSSION

Paf1 and its associated proteins in Paf1C likely act as a platform, recruiting factors that connect Paf1 to multiple transcription processes from elongation to polyadenylation and termination (Chen et al., 2015; Tomson and Arndt, 2013; Yang et al., 2016; Yu et al., 2015). Given Paf1C's role in mediating post-translational modifications to histones, such as H2Bub, and its capacity to form protein:protein interactions with factors involved in RNA processing and nuclear export (Table S4), it is not surprising that Paf1 has additional roles in regulating nuclear export of transcripts, as we show here. What is surprising is that this is a selective event and distinct from Paf1's role in elongation. Given the biochemical evidence that Paf1's function in elongation is direct (Rondón et al., 2004; Tous et al., 2011), we propose that there may be two distinct Paf1-associated functions. Low levels of Paf1, equal to that on the most Paf1-depleted gene defined using TEF-seq, would be sufficient for the core elongation function on all genes. The second function would result from differential association of Paf1 with Pol2 above this level, leading to selective promotion of RNA nuclear export. Our analysis implicates the promoter as the main feature linked to enrichment of Paf1 on Pol2 transcribing the associated gene. Furthermore, levels of Ser2-phosphorylated residues with the CTD, only when neighboring Ser5 residues are also phosphorylated, maintain differential enrichment or depletion during transcription elongation. There are a number of possible ways to envisage how the promoter is linked to Paf1 enrichment on Pol2, all involving specific DNA sequence elements and transcription factors such as Sfp1, Haa1, or Cbf1. Transcription factors (TFs) are known to influence the transition from a more Ser5- to a more Ser2-phosphorylated CTD during transcription elongation (Kwak and Lis, 2013). By analogy with the role of MYC in mammalian cells (Jaenicke et al., 2016), TFs could recruit Paf1, perhaps in association with another factor, such as CK2, known to interact with both Paf1 and Sfp1, and then transfer Paf1 to Pol2. Alternatively, TFs could recruit CTD kinases such as Bur1 (CDK9) or Ctk1 (CDK12), leading to differential Ser2 phosphorylation levels on Pol2 and, hence, differential Paf1 levels. Finally, TFs could promote recruitment of a Paf1-enriched pre-initiation complex (PIC) as opposed to a mediator-enriched PIC (Hampsey, 1998).

Current models propose that transcript fate is determined by poly(A) tail length and the proteins associated with different transcripts during 3′ end formation (Tuck and Tollervey, 2013). However, we show here that transcript poly(A) tail length (Subtelny et al., 2014) showed no relationship to Paf1 enrichment or depletion on mRNA genes (Figure S5) and, thus, is unlikely to be related to Paf1's role in selectively promoting transcript nuclear export. Instead, our data suggest that the proteins associated with these transcripts, and thus their fate, can be determined before 3′ end formation and is related to the levels of Paf1 in the elongation complex during transcription elongation, which is dynamic and subject to environmental control via promoters. Transcript fate can be modulated by signals received at the promoter, already shown to influence transcript localization in the cytoplasm (Zid and O'Shea, 2014) and half-life (Bregman et al., 2011; Trcek et al., 2011). Our data add differential RNA export to the functions likely to be determined by the promoter.

When Paf1 is associated with Pol2, we envisage two consequences. Paf1C is required for H2Bub. H2Bub stimulates the ubiquitylation of the associated with the Pta1 subunit of CPF (APT) complex subunit Swd2, which, in turn, causes an association between Mex67 and Swd2 and independently facilitates recruitment of the Mex67 adaptor Npl3 to nascent transcripts to promote nuclear export (Vitaliano-Prunier et al., 2012). Consistently, genes and transcription units with Pol2 depleted for Paf1 also show reduced H2Bub (Murray et al., 2015). Alternatively, Paf1C could simply be transported with Pol2 to the 3′ region to recruit or modify RNA binding and processing enzymes, such as Nab2, Npl3, Yra1, and Hpr1. Paf1 interacts with Hpr1 (Chang et al., 1999), which, in turn, interacts with Mex67 and Yra1, facilitating interactions with the nuclear pore. Hpr1 also undergoes sumoylation, enabling it to bind specific RNAs, including members of the *HAA1* regulon (Bretes et al., 2014), shown here to be Paf1-enriched.

NET-seq supports a role for Paf1 in the control of transcription elongation. Paf1 changes the relative distribution of Pol2 over the 5′ and 3′ regions of genes. Paf1 deletion in yeast and knockdown in mammals results in increased Pol2 CTD phosphorylation at Ser5 and reduced phosphorylation at Ser2 (Mueller et al., 2004; Nordick et al., 2008; Yu et al., 2015), although, in other work, an increase in Ser2 phosphorylation is observed (Chen et al., 2015). The disruption of Paf1-dependent CTD phosphorylation patterns could contribute to or result from slower elongation (Tomson and Arndt, 2013) and compromised checkpoints (Laitem et al., 2015; Rodríguez-Molina et al., 2016), leading to more Pol2 on the body of the gene, and supports some conservation of Paf1's role in controlling transcription elongation across the species. However, Paf1's role in transcription elongation is not related to the differences in Paf1 levels on Pol2 observed at different genes.

In summary, our findings support distinct forms of the Pol2 elongation complex, with a core function in elongation and a regulatory function determining differential transcript fate, determined by levels of Paf1 on Pol2. Paf1 is unlikely to be unique in showing enrichment or depletion on elongating Pol2; preliminary data show enrichment or depletion of other elongation factors on

(C) Bar charts showing the mean ratio of nuclear to whole-cell transcript levels in glucose and after 60 min in galactose for the loci shown, measured using qRT-PCR for transcripts encoded by the genes shown above. Transcript levels are normalized to the level of the *CLB1* transcript in each sample. Error bars show the SEM p values (Welch's t test) calculated to test the significance of the increase in the mean ratio in galactose (n = 4).
See also Tables S3 and S6.

Pol2 at different groups of genes. The use of an IP step in TEF-seq makes it eminently adaptable to studying a range of different Pol2-associated factors in different organisms, cell types, or growth conditions and to enhance our understanding of transcription elongation and its relationship to transcript fate. It will be important to determine the conservation of Paf1's role in transcript fate in metazoans, and development of TEF-seq in these systems will enable this. We anticipate that, given the degree of overlapping pervasive transcription in eukaryotic genomes (Mellor et al., 2016; Murray and Mellor, 2016), the strand-specific nucleotide resolution of TEF-seq will be an invaluable tool.

STAR★METHODS

Detailed methods are provided in the online version of this paper and include the following:

- KEY RESOURCES TABLE
- CONTACT FOR REAGENT AND RESOURCE SHARING
- EXPERIMENTAL MODEL AND SUBJECT DETAILS
- METHOD DETAIL
  - Genetic manipulation of yeast strains
  - Biochemistry
- QUANTIFICATION AND STATISTICAL ANALYSIS
  - NET-seq/TEF-seq sequencing data processing
  - RNA FISH analysis
- DATA AND SOFTWARE AVAILABILITY

SUPPLEMENTAL INFORMATION

Supplemental Information includes five figures and six tables and can be found with this article online at http://dx.doi.org/10.1016/j.molcel.2017.01.006.

AUTHOR CONTRIBUTIONS

H.F. and J.M. designed the experiments. H.F and F.S.H. performed the experiments and analyzed the data. H.F. and J.M. wrote the manuscript. H.F., F.H., A.F., and J.M. revised the manuscript.

ACKNOWLEDGMENTS

We thank Nick Proudfoot, Neil Brockdorff, Patrick Cramer, Alexandre Akoulitchev, and the J.M. lab for critical discussions, Anitha Nair for excellent technical support, and Ilan Davis and Micron Oxford for microscopy support. This work was supported by The Wellcome Trust (WT089156MA to J.M.), a Wellcome Trust studentship (097302/Z/11/Z to H.F.), the Biotechnology and Biological Sciences Research Council (BB/N001184/1 to A.F.), and a Wellcome Trust Strategic Award (091911) supporting advanced microscopy at Micron Oxford (http://micronoxford.com). J.M. acts as an advisor to and holds stock in Oxford Biodynamics plc., Chronos Therapeutics Ltd., and Sibelius Natural Products Ltd.

Received: March 14, 2016
Revised: September 22, 2016
Accepted: January 5, 2017
Published: February 9, 2017

SUPPORTING CITATIONS

The following references appear in the Supplemental Information: Cho et al. (1998); Dichtl et al. (2002); Fernandes et al. (2005); Hausmann et al. (2005); Jaehning (2010); Kim and Levin (2011); Kim et al. (2004); Kim et al. (2010); Krishnamurthy et al. (2004); Krogan et al. (2002); Lidschreiber et al. (2013); Liu et al. (2011); Lunde et al. (2010); Malone et al. (1991); Martinez-Rucobo et al. (2015); Mayer et al. (2012); Meinhart and Cramer (2004); Noble et al. (2005); Pei et al. (2001); Rosado-Lugo and Hampsey (2014); Shi et al. (1996); Sun et al. (2010); Vojnic et al. (2006); Werner-Allen et al. (2011); Winkler et al. (2011); Xiao et al. (2003); Youdell et al. (2008); Zhang et al. (2012).

REFERENCES

Baejen, C., Torkler, P., Gressel, S., Essig, K., Söding, J., and Cramer, P. (2014). Transcriptome maps of mRNP biogenesis factors define pre-mRNA recognition. Mol. Cell *55*, 745–757.

Bailey, T.L. (2011). DREME: motif discovery in transcription factor ChIP-seq data. Bioinformatics *27*, 1653–1659.

Blankenberg, D., Von Kuster, G., Coraor, N., Ananda, G., Lazarus, R., Mangan, M., Nekrutenko, A., and Taylor, J. (2010). Galaxy: a web-based genome analysis tool for experimentalists. Curr. Protoc. Mol. Biol. *Chapter 19*. Unit 19 10 11–21.

Bortvin, A., and Winston, F. (1996). Evidence that Spt6p controls chromatin structure by a direct interaction with histones. Science *272*, 1473–1476.

Bregman, A., Avraham-Kelbert, M., Barkai, O., Duek, L., Guterman, A., and Choder, M. (2011). Promoter elements regulate cytoplasmic mRNA decay. Cell *147*, 1473–1483.

Bretes, H., Rouviere, J.O., Leger, T., Oeffinger, M., Devaux, F., Doye, V., and Palancade, B. (2014). Sumoylation of the THO complex regulates the biogenesis of a subset of mRNPs. Nucleic Acids Res. *42*, 5043–5058.

Brogaard, K., Xi, L., Wang, J.P., and Widom, J. (2012). A map of nucleosome positions in yeast at base-pair resolution. Nature *486*, 496–501.

Chang, M., French-Cornay, D., Fan, H.Y., Klein, H., Denis, C.L., and Jaehning, J.A. (1999). A complex containing RNA polymerase II, Paf1p, Cdc73p, Hpr1p, and Ccr4p plays a role in protein kinase C signaling. Mol. Cell. Biol. *19*, 1056–1067.

Chen, F.X., Woodfin, A.R., Gardini, A., Rickels, R.A., Marshall, S.A., Smith, E.R., Shiekhattar, R., and Shilatifard, A. (2015). PAF1, a Molecular Regulator of Promoter-Proximal Pausing by RNA Polymerase II. Cell *162*, 1003–1015.

Cho, E.J., Rodriguez, C.R., Takagi, T., and Buratowski, S. (1998). Allosteric interactions between capping enzyme subunits and the RNA polymerase II carboxy-terminal domain. Genes Dev. *12*, 3482–3487.

Churchman, L.S., and Weissman, J.S. (2011). Nascent transcript sequencing visualizes transcription at nucleotide resolution. Nature *469*, 368–373.

Custódio, N., and Carmo-Fonseca, M. (2016). Co-transcriptional splicing and the CTD code. Crit. Rev. Biochem. Mol. Biol. *51*, 395–411.

Dermody, J.L., and Buratowski, S. (2010). Leo1 subunit of the yeast paf1 complex binds RNA and contributes to complex recruitment. J. Biol. Chem. *285*, 33671–33679.

Dichtl, B., Blank, D., Ohnacker, M., Friedlein, A., Roeder, D., Langen, H., and Keller, W. (2002). A role for SSU72 in balancing RNA polymerase II transcription elongation and termination. Mol. Cell *10*, 1139–1150.

Dronamraju, R., and Strahl, B.D. (2014). A feed forward circuit comprising Spt6, Ctk1 and PAF regulates Pol II CTD phosphorylation and transcription elongation. Nucleic Acids Res. *42*, 870–881.

Fernandes, A.R., Mira, N.P., Vargas, R.C., Canelhas, I., and Sá-Correia, I. (2005). Saccharomyces cerevisiae adaptation to weak acids involves the transcription factor Haa1p and Haa1p-regulated genes. Biochem. Biophys. Res. Commun. *337*, 95–103.

Hampsey, M. (1998). Molecular genetics of the RNA polymerase II general transcriptional machinery. Microbiol. Mol. Biol. Rev. *62*, 465–503.

Hausmann, S., Koiwa, H., Krishnamurthy, S., Hampsey, M., and Shuman, S. (2005). Different strategies for carboxyl-terminal domain (CTD) recognition by serine 5-specific CTD phosphatases. J. Biol. Chem. *280*, 37681–37688.

Hieronymus, H., and Silver, P.A. (2003). Genome-wide analysis of RNA-protein interactions illustrates specificity of the mRNA export machinery. Nat. Genet. *33*, 155–161.

Hondele, M., Stuwe, T., Hassler, M., Halbach, F., Bowman, A., Zhang, E.T., Nijmeijer, B., Kotthoff, C., Rybin, V., Amlacher, S., et al. (2013). Structural basis of histone H2A-H2B recognition by the essential chaperone FACT. Nature *499*, 111–114.

Hu, Z., Killion, P.J., and Iyer, V.R. (2007). Genetic reconstruction of a functional transcriptional regulatory network. Nat. Genet. *39*, 683–687.

Jaehning, J.A. (2010). The Paf1 complex: platform or player in RNA polymerase II transcription? Biochim. Biophys. Acta *1799*, 379–388.

Jaenicke, L.A., von Eyss, B., Carstensen, A., Wolf, E., Xu, W., Greifenberg, A.K., Geyer, M., Eilers, M., and Popov, N. (2016). Ubiquitin-Dependent Turnover of MYC Antagonizes MYC/PAF1C Complex Accumulation to Drive Transcriptional Elongation. Mol. Cell *61*, 54–67.

Jiang, C., and Pugh, B.F. (2009). Nucleosome positioning and gene regulation: advances through genomics. Nat. Rev. Genet. *10*, 161–172.

Jorgensen, P., Nishikawa, J.L., Breitkreutz, B.J., and Tyers, M. (2002). Systematic identification of pathways that couple cell growth and division in yeast. Science *297*, 395–400.

Keller, G., Ray, E., Brown, P.O., and Winge, D.R. (2001). Haa1, a protein homologous to the copper-regulated transcription factor Ace1, is a novel transcriptional activator. J. Biol. Chem. *276*, 38697–38702.

Kim, K.Y., and Levin, D.E. (2011). Mpk1 MAPK association with the Paf1 complex blocks Sen1-mediated premature transcription termination. Cell *144*, 745–756.

Kim, M., Ahn, S.H., Krogan, N.J., Greenblatt, J.F., and Buratowski, S. (2004). Transitions in RNA polymerase II elongation complexes at the 3′ ends of genes. EMBO J. *23*, 354–364.

Kim, H., Erickson, B., Luo, W., Seward, D., Graber, J.H., Pollock, D.D., Megee, P.C., and Bentley, D.L. (2010). Gene-specific RNA polymerase II phosphorylation and the CTD code. Nat. Struct. Mol. Biol. *17*, 1279–1286.

Kizer, K.O., Phatnani, H.P., Shibata, Y., Hall, H., Greenleaf, A.L., and Strahl, B.D. (2005). A novel domain in Set2 mediates RNA polymerase II interaction and couples histone H3 K36 methylation with transcript elongation. Mol. Cell. Biol. *25*, 3305–3316.

Kowalik, K.M., Shimada, Y., Flury, V., Stadler, M.B., Batki, J., and Bühler, M. (2015). The Paf1 complex represses small-RNA-mediated epigenetic gene silencing. Nature *520*, 248–252.

Krishnamurthy, S., He, X., Reyes-Reyes, M., Moore, C., and Hampsey, M. (2004). Ssu72 Is an RNA polymerase II CTD phosphatase. Mol. Cell *14*, 387–394.

Krogan, N.J., Kim, M., Ahn, S.H., Zhong, G., Kobor, M.S., Cagney, G., Emili, A., Shilatifard, A., Buratowski, S., and Greenblatt, J.F. (2002). RNA polymerase II elongation factors of Saccharomyces cerevisiae: a targeted proteomics approach. Mol. Cell. Biol. *22*, 6979–6992.

Krogan, N.J., Peng, W.T., Cagney, G., Robinson, M.D., Haw, R., Zhong, G., Guo, X., Zhang, X., Canadien, V., Richards, D.P., et al. (2004). High-definition macromolecular composition of yeast RNA-processing complexes. Mol. Cell *13*, 225–239.

Kwak, H., and Lis, J.T. (2013). Control of transcriptional elongation. Annu. Rev. Genet. *47*, 483–508.

Laitem, C., Zaborowska, J., Isa, N.F., Kufs, J., Dienstbier, M., and Murphy, S. (2015). CDK9 inhibitors define elongation checkpoints at both ends of RNA polymerase II-transcribed genes. Nat. Struct. Mol. Biol. *22*, 396–403.

Langmead, B. (2010). Aligning short sequencing reads with Bowtie. Curr. Protoc. Bioinformatics *Chapter 11*. Unit 11.7.

Li, H., Handsaker, B., Wysoker, A., Fennell, T., Ruan, J., Homer, N., Marth, G., Abecasis, G., and Durbin, R.; 1000 Genome Project Data Processing Subgroup (2009). The Sequence Alignment/Map format and SAMtools. Bioinformatics *25*, 2078–2079.

Lidschreiber, M., Leike, K., and Cramer, P. (2013). Cap completion and C-terminal repeat domain kinase recruitment underlie the initiation-elongation transition of RNA polymerase II. Mol. Cell. Biol. *33*, 3805–3816.

Liu, J., Zhang, J., Gong, Q., Xiong, P., Huang, H., Wu, B., Lu, G., Wu, J., and Shi, Y. (2011). Solution structure of tandem SH2 domains from Spt6 protein and their binding to the phosphorylated RNA polymerase II C-terminal domain. J. Biol. Chem. *286*, 29218–29226.

Longtine, M.S., McKenzie, A., 3rd, Demarini, D.J., Shah, N.G., Wach, A., Brachat, A., Philippsen, P., and Pringle, J.R. (1998). Additional modules for versatile and economical PCR-based gene deletion and modification in *Saccharomyces cerevisiae*. Yeast *14*, 953–961.

Love, M.I., Huber, W., and Anders, S. (2014). Moderated estimation of fold change and dispersion for RNA-seq data with DESeq2. Genome Biol. *15*, 550.

Lunde, B.M., Reichow, S.L., Kim, M., Suh, H., Leeper, T.C., Yang, F., Mutschler, H., Buratowski, S., Meinhart, A., and Varani, G. (2010). Cooperative interaction of transcription termination factors with the RNA polymerase II C-terminal domain. Nat. Struct. Mol. Biol. *17*, 1195–1201.

Mahony, S., and Pugh, B.F. (2015). Protein-DNA binding in high-resolution. Crit. Rev. Biochem. Mol. Biol. *50*, 269–283.

Malone, E.A., Clark, C.D., Chiang, A., and Winston, F. (1991). Mutations in SPT16/CDC68 suppress cis- and trans-acting mutations that affect promoter function in Saccharomyces cerevisiae. Mol. Cell. Biol. *11*, 5710–5717.

Martinez-Rucobo, F.W., Kohler, R., van de Waterbeemd, M., Heck, A.J., Hemann, M., Herzog, F., Stark, H., and Cramer, P. (2015). Molecular Basis of Transcription-Coupled Pre-mRNA Capping. Mol. Cell *58*, 1079–1089.

Mayer, A., Lidschreiber, M., Siebert, M., Leike, K., Söding, J., and Cramer, P. (2010). Uniform transitions of the general RNA polymerase II transcription complex. Nat. Struct. Mol. Biol. *17*, 1272–1278.

Mayer, A., Heidemann, M., Lidschreiber, M., Schreieck, A., Sun, M., Hintermair, C., Kremmer, E., Eick, D., and Cramer, P. (2012). CTD tyrosine phosphorylation impairs termination factor recruitment to RNA polymerase II. Science *336*, 1723–1725.

Meinhart, A., and Cramer, P. (2004). Recognition of RNA polymerase II carboxy-terminal domain by 3′-RNA-processing factors. Nature *430*, 223–226.

Mellor, J., Jiang, W., Funk, M., Rathjen, J., Barnes, C.A., Hinz, T., Hegemann, J.H., and Philippsen, P. (1990). CPF1, a yeast protein which functions in centromeres and promoters. EMBO J. *9*, 4017–4026.

Mellor, J., Woloszczuk, R., and Howe, F.S. (2016). The Interleaved Genome. Trends Genet. *32*, 57–71.

Milligan, L., Huynh-Thu, V.A., Delan-Forino, C., Tuck, A., Petfalski, E., Lombraña, R., Sanguinetti, G., Kudla, G., and Tollervey, D. (2016). Strand-specific, high-resolution mapping of modified RNA polymerase II. Mol. Syst. Biol. *12*, 874.

Mueller, C.L., Porter, S.E., Hoffman, M.G., and Jaehning, J.A. (2004). The Paf1 complex has functions independent of actively transcribing RNA polymerase II. Mol. Cell *14*, 447–456.

Murray, S.C., and Mellor, J. (2016). Using both strands: The fundamental nature of antisense transcription. BioArchitecture *6*, 12–21.

Murray, S.C., Haenni, S., Howe, F.S., Fischl, H., Chocian, K., Nair, A., and Mellor, J. (2015). Sense and antisense transcription are associated with distinct chromatin architectures across genes. Nucleic Acids Res. *43*, 7823–7837.

Murray, S.C., Serra Barros, A., Brown, D.A., Dudek, P., Ayling, J., and Mellor, J. (2012). A pre-initiation complex at the 3′-end of genes drives antisense transcription independent of divergent sense transcription. Nucleic Acids Res. *40*, 2432–2444.

Nguyen, T., Fischl, H., Howe, F.S., Woloszczuk, R., Serra Barros, A., Xu, Z., Brown, D., Murray, S.C., Haenni, S., Halstead, J.M., et al. (2014). Transcription mediated insulation and interference direct gene cluster expression switches. eLife *3*, e03635.

Noble, C.G., Hollingworth, D., Martin, S.R., Ennis-Adeniran, V., Smerdon, S.J., Kelly, G., Taylor, I.A., and Ramos, A. (2005). Key features of the interaction

between Pcf11 CID and RNA polymerase II CTD. Nat. Struct. Mol. Biol. *12*, 144–151.

Nordick, K., Hoffman, M.G., Betz, J.L., and Jaehning, J.A. (2008). Direct interactions between the Paf1 complex and a cleavage and polyadenylation factor are revealed by dissociation of Paf1 from RNA polymerase II. Eukaryot. Cell *7*, 1158–1167.

Otsu, N. (1979). A Threshold Selection Method from Gray-Level Histograms. Systems, Man and Cybernetics. IEEE Transactions on *9*, 62–66.

Pei, Y., Hausmann, S., Ho, C.K., Schwer, B., and Shuman, S. (2001). The length, phosphorylation state, and primary structure of the RNA polymerase II carboxyl-terminal domain dictate interactions with mRNA capping enzymes. J. Biol. Chem. *276*, 28075–28082.

Pelechano, V., Wei, W., and Steinmetz, L.M. (2013). Extensive transcriptional heterogeneity revealed by isoform profiling. Nature *497*, 127–131.

Poli, J., Gerhold, C.B., Tosi, A., Hustedt, N., Seeber, A., Sack, R., Herzog, F., Pasero, P., Shimada, K., Hopfner, K.P., and Gasser, S.M. (2016). Mec1, INO80, and the PAF1 complex cooperate to limit transcription replication conflicts through RNAPII removal during replication stress. Genes Dev. *30*, 337–354.

Qiu, H., Hu, C., Gaur, N.A., and Hinnebusch, A.G. (2012). Pol II CTD kinases Bur1 and Kin28 promote Spt5 CTR-independent recruitment of Paf1 complex. EMBO J. *31*, 3494–3505.

Rodríguez-Molina, J.B., Tseng, S.C., Simonett, S.P., Taunton, J., and Ansari, A.Z. (2016). Engineered Covalent Inactivation of TFIIH-Kinase Reveals an Elongation Checkpoint and Results in Widespread mRNA Stabilization. Mol. Cell *63*, 433–444.

Rondón, A.G., Gallardo, M., García-Rubio, M., and Aguilera, A. (2004). Molecular evidence indicating that the yeast PAF complex is required for transcription elongation. EMBO Rep. *5*, 47–53.

Rosado-Lugo, J.D., and Hampsey, M. (2014). The Ssu72 phosphatase mediates the RNA polymerase II initiation-elongation transition. J. Biol. Chem. *289*, 33916–33926.

Schindelin, J., Arganda-Carreras, I., Frise, E., Kaynig, V., Longair, M., Pietzsch, T., Preibisch, S., Rueden, C., Saalfeld, S., Schmid, B., et al. (2012). Fiji: an open-source platform for biological-image analysis. Nat. Methods *9*, 676–682.

Shi, X., Finkelstein, A., Wolf, A.J., Wade, P.A., Burton, Z.F., and Jaehning, J.A. (1996). Paf1p, an RNA polymerase II-associated factor in Saccharomyces cerevisiae, may have both positive and negative roles in transcription. Mol. Cell. Biol. *16*, 669–676.

Squazzo, S.L., Costa, P.J., Lindstrom, D.L., Kumer, K.E., Simic, R., Jennings, J.L., Link, A.J., Arndt, K.M., and Hartzog, G.A. (2002). The Paf1 complex physically and functionally associates with transcription elongation factors in vivo. EMBO J. *21*, 1764–1774.

Subtelny, A.O., Eichhorn, S.W., Chen, G.R., Sive, H., and Bartel, D.P. (2014). Poly(A)-tail profiling reveals an embryonic switch in translational control. Nature *508*, 66–71.

Sun, M., Larivière, L., Dengl, S., Mayer, A., and Cramer, P. (2010). A tandem SH2 domain in transcription elongation factor Spt6 binds the phosphorylated RNA polymerase II C-terminal repeat domain (CTD). J. Biol. Chem. *285*, 41597–41603.

Thorvaldsdóttir, H., Robinson, J.T., and Mesirov, J.P. (2013). Integrative Genomics Viewer (IGV): high-performance genomics data visualization and exploration. Brief. Bioinform. *14*, 178–192.

Tomson, B.N., and Arndt, K.M. (2013). The many roles of the conserved eukaryotic Paf1 complex in regulating transcription, histone modifications, and disease states. Biochim. Biophys. Acta *1829*, 116–126.

Tous, C., Rondón, A.G., García-Rubio, M., González-Aguilera, C., Luna, R., and Aguilera, A. (2011). A novel assay identifies transcript elongation roles for the Nup84 complex and RNA processing factors. EMBO J. *30*, 1953–1964.

Trcek, T., Larson, D.R., Moldón, A., Query, C.C., and Singer, R.H. (2011). Single-molecule mRNA decay measurements reveal promoter- regulated mRNA stability in yeast. Cell *147*, 1484–1497.

Tuck, A.C., and Tollervey, D. (2013). A transcriptome-wide atlas of RNP composition reveals diverse classes of mRNAs and lncRNAs. Cell *154*, 996–1009.

Villanyi, Z., Ribaud, V., Kassem, S., Panasenko, O.O., Pahi, Z., Gupta, I., Steinmetz, L., Boros, I., and Collart, M.A. (2014). The Not5 subunit of the ccr4-not complex connects transcription and translation. PLoS Genet. *10*, e1004569.

Vitaliano-Prunier, A., Babour, A., Hérissant, L., Apponi, L., Margaritis, T., Holstege, F.C., Corbett, A.H., Gwizdek, C., and Dargemont, C. (2012). H2B ubiquitylation controls the formation of export-competent mRNP. Mol. Cell *45*, 132–139.

Vojnic, E., Simon, B., Strahl, B.D., Sattler, M., and Cramer, P. (2006). Structure and carboxyl-terminal domain (CTD) binding of the Set2 SRI domain that couples histone H3 Lys36 methylation to transcription. J. Biol. Chem. *281*, 13–15.

Werner-Allen, J.W., Lee, C.J., Liu, P., Nicely, N.I., Wang, S., Greenleaf, A.L., and Zhou, P. (2011). cis-Proline-mediated Ser(P)5 dephosphorylation by the RNA polymerase II C-terminal domain phosphatase Ssu72. J. Biol. Chem. *286*, 5717–5726.

Wickramasinghe, V.O., and Laskey, R.A. (2015). Control of mammalian gene expression by selective mRNA export. Nat. Rev. Mol. Cell Biol. *16*, 431–442.

Winkler, D.D., Muthurajan, U.M., Hieb, A.R., and Luger, K. (2011). Histone chaperone FACT coordinates nucleosome interaction through multiple synergistic binding events. J. Biol. Chem. *286*, 41883–41892.

Xiao, T., Hall, H., Kizer, K.O., Shibata, Y., Hall, M.C., Borchers, C.H., and Strahl, B.D. (2003). Phosphorylation of RNA polymerase II CTD regulates H3 methylation in yeast. Genes Dev. *17*, 654–663.

Yan, C., Zhang, D., Raygoza Garay, J.A., Mwangi, M.M., and Bai, L. (2015). Decoupling of divergent gene regulation by sequence-specific DNA binding factors. Nucleic Acids Res. *43*, 7292–7305.

Yang, Y., Li, W., Hoque, M., Hou, L., Shen, S., Tian, B., and Dynlacht, B.D. (2016). PAF Complex Plays Novel Subunit-Specific Roles in Alternative Cleavage and Polyadenylation. PLoS Genet. *12*, e1005794.

Youdell, M.L., Kizer, K.O., Kisseleva-Romanova, E., Fuchs, S.M., Duro, E., Strahl, B.D., and Mellor, J. (2008). Roles for Ctk1 and Spt6 in regulating the different methylation states of histone H3 lysine 36. Mol. Cell. Biol. *28*, 4915–4926.

Yu, M., Yang, W., Ni, T., Tang, Z., Nakadai, T., Zhu, J., and Roeder, R.G. (2015). RNA polymerase II-associated factor 1 regulates the release and phosphorylation of paused RNA polymerase II. Science *350*, 1383–1386.

Zhang, D.W., Mosley, A.L., Ramisetty, S.R., Rodríguez-Molina, J.B., Washburn, M.P., and Ansari, A.Z. (2012). Ssu72 phosphatase-dependent erasure of phospho-Ser7 marks on the RNA polymerase II C-terminal domain is essential for viability and transcription termination. J. Biol. Chem. *287*, 8541–8551.

Zid, B.M., and O'Shea, E.K. (2014). Promoter sequences direct cytoplasmic localization and translation of mRNAs during starvation in yeast. Nature *514*, 117–121.

STAR★METHODS

KEY RESOURCES TABLE

REAGENT or RESOURCE	SOURCE	IDENTIFIER
Antibodies		
Mouse monoclonal anti FLAG-tag peptide sequence (clone M2) (Dilution for western blot: 1:5000)	Sigma-Aldrich	Cat#F1804, RRID: AB_262044
Rat monoclonal anti HA-tag peptide sequence (clone 3F10) (Dilution for western blot: 1:500)	Roche	Cat#ROAHAHA
Rabbit monoclonal anti residues 1-80 from N terminus of Rpb1 (clone σ80) (Dilution for western blot: 7:1000)	Santa Cruz	Cat#sc-25758, RRID: AB_655813
Rat monoclonal anti-Ser2P Rpb1 CTD (clone 3E10) (Dilution for western blot: 1:1000)	Millipore	Cat#04-1571, RRID: AB_11212363
Rat monoclonal anti-Ser5P Rpb1 CTD (clone 3E8) (Dilution for western blot: 1:2000)	Millipore	Cat#04-1572, RRID: AB_10615822
Rat monoclonal anti-Ser7P Rpb1 CTD (clone 4E12) (Dilution for western blot: 1:2000)	Millipore	Cat#04-1570, RRID: AB_10618152
Chemicals, Peptides, and Recombinant Proteins		
Anti-FLAG M2 affinity agarose beads (affinity gel)	Sigma-Aldrich	Cat#A2220
Anti-HA Dynabeads	Pierce	Cat#88837
3 × FLAG peptide	Sigma-Aldrich	Cat#F4799
Critical Commercial Assays		
miRNeasy kit	QIAGEN	Cat#217004
Deposited Data		
Raw and processed data	This paper	ArrayExpress: E-MTAB-4568
Images and data	This paper	Mendeley: DOI: 10.17632/2vhybtrwvk.1
Annotations of most abundant mRNA and lncRNA transcript isoforms	Pelechano et al., 2013	GEO: GSE39128
Annotations of introns and non-coding RNAs	Saccharomyces Genome Database (SGD)	http://www.yeastgenome.org/
S. cerevisiae reference genome: sacCer3 (April 2011 sequence)	SGD	http://www.yeastgenome.org/
Rpb3 ChIP-chip processed data	Mayer et al., 2010	ArrayExpress: E-TABM-1033
Processed data	Baejen et al., 2014	GEO: GSE59676
Gene lists from clustering analysis	Tuck and Tollervey, 2013	N/A
Experimental Models: Organisms/Strains		
S. cerevisiae: Name = BY4741; Genotype = MATa; his3Δ1; leu2Δ0; met15Δ0; ura3Δ0	Euroscarf	N/A
S. cerevisiae: Name = YSC001; Genotype = RPB3-3xFLAG-NAT	Churchman and Weissman, 2011	N/A
S. cerevisiae: Name = BY4741 *RPB3-3xFLAG;* Genotype = *RPB3-3xFLAG-His3MX6*	This paper	N/A
S. cerevisiae: Name = BY4741 *RPB2-3xFLAG;* Genotype = *RPB2-3xFLAG-His3MX6*	This paper	N/A
S. cerevisiae: Name = BY4741 *SPT6-3xFLAG;* Genotype = *SPT6-3xFLAG-His3MX6*	This paper	N/A
S. cerevisiae: Name = BY4741 *SPT16-3xFLAG;* Genotype = *SPT16-3xFLAG-His3MX6*	This paper	N/A
S. cerevisiae: Name = BY4741 *PAF1-3xFLAG;* Genotype = *PAF1-3xFLAG-His3MX6*	This paper	N/A
S. cerevisiae: Name = BY4741 *PCF11-3xFLAG;* Genotype = *PCF11-3xFLAG-His3MX6*	This paper	N/A

(*Continued on next page*)

Continued

REAGENT or RESOURCE	SOURCE	IDENTIFIER
S. cerevisiae: Name = BY4741 *CET1-3xFLAG;* Genotype = *CET1-3xFLAG-His3MX6*	This paper	N/A
S. cerevisiae: Name = BY4741 SSU72-*3xFLAG;* Genotype = *SSU72-3xFLAG-His3MX6*	This paper	N/A
S. cerevisiae: Name = BY4742 SET2-*3xFLAG;* Genotype = *SET2-3xFLAG-His3MX6*	This paper	N/A
S. cerevisiae: Name = BY4741 *RPB3-3xFLAG PAF1-3xHA;* Genotype = *RPB3-3xFLAG-His3MX6, PAF1-3xHA-KanMX6*	This paper	N/A
S. cerevisiae: Name = BY4741 *RPB3-3xFLAG RPB2-3xHA;* Genotype = *RPB3-3xFLAG-His3MX6, RPB2-3xHA-KanMX6*	This paper	N/A
S. cerevisiae: Name = BY4741 *RPB3-3xFLAG paf1Δ;* Genotype = *RPB3-3xFLAG-HisMX6, paf1::KanMX6*	This paper	N/A
S. cerevisiae: Name = BY4741 *paf1Δ;* Genotype = *paf1Δ::KanMX6*	This paper	N/A
S. cerevisiae: Name = BY4741 *slc1::URA3*; Genotype = *slc1::URA3*	This paper	N/A
S. cerevisiae: Name = BY4741 *slc1::URA3 RPB3-3xFLAG*; Genotype = *slc1::URA3, RPB3-3xFLAG-His3MX6*	This paper	N/A
S. cerevisiae: Name = BY4741 *slc1::URA3 PAF1-3xFLAG*; Genotype = *slc1::URA3, PAF1-3xFLAG-His3MX6*	This paper	N/A
S. cerevisiae: Name = BY4741 *cmk2::URA3*; Genotype = *cmk2::URA3*	This paper	N/A
S. cerevisiae: Name = BY4741 *cmk2::URA3 RPB3-3xFLAG*; Genotype = *cmk2::URA3, RPB3-3xFLAG-His3MX6*	This paper	N/A
S. cerevisiae: Name = BY4741 *cmk2::URA3 PAF1-3xFLAG*; Genotype = *cmk2::URA3, PAF1-3xFLAG-His3MX6*	This paper	N/A
S. cerevisiae: Name = BY4741 *GAL1::ADH1*$_T$; see Murray et al. (2012) for genotype details	Murray et al., 2012	N/A
S. cerevisiae: Name = BY4741 *GAL1::ADH1*$_T$ *paf1Δ*; Genotype = BY4741 *GAL1::ADH1*$_T$, *paf1::KanMX6*	This paper	N/A
Sequence-Based Reagents		
RT primer: 5′/5Phos/ATCTCGTATGCCGTCTTCTGCTTG/iSp18/CACTCA/iSp18/TCCGACGATCATTGATGGTGCCTACAG 3′	IDT	Custom order
Barcoded primer 1: AATGATACGGCGACCACCGAGATCTACACGATCGGAAGAGCACACGTCTGAACTCCAGTCACATGCCATCCGACGATCATTGATGG	IDT	Custom order
Barcoded primer 2: AATGATACGGCGACCACCGAGATCTACACGATCGGAAGAGCACACGTCTGAACTCCAGTCACTGCATCTCCGACGATCATTGATGG	IDT	Custom order
Barcoded primer 3: AATGATACGGCGACCACCGAGATCTACACGATCGGAAGAGCACACGTCTGAACTCCAGTCACTTAGGCTCCGACGATCATTGATGG	IDT	Custom order
Barcoded primer 4: AATGATACGGCGACCACCGAGATCTACACGATCGGAAGAGCACACGTCTGAACTCCAGTCACTGACCATCCGACGATCATTGATGG	IDT	Custom order
Primer 1: CAAGCAGAAGACGGCATACGA	IDT	Custom order
Sequencing primer: TCCGACGATCATTGATGGTGCCTACAG	IDT	Custom order
Primers for reverse transcription PCR. See Table S5	N/A	N/A
RNA FISH probes. See Table S6	Biosearch Technologies	Custom order
Software and Algorithms		
Galaxy	Blankenberg et al., 2010	https://usegalaxy.org
Bowtie for Illumina	Langmead, 2010	https://usegalaxy.org
Samtools	Li et al., 2009	https://usegalaxy.org
Integrated Genome Viewer	Thorvaldsdóttir et al., 2013	http://software.broadinstitute.org/software/igv/

(*Continued on next page*)

Continued

REAGENT or RESOURCE	SOURCE	IDENTIFIER
R/Bioconductor	Bioconductor	https://www.bioconductor.org/
DREME	Bailey, 2011	http://meme-suite.org/
DESeq2	Love et al., 2014	https://bioconductor.org/packages/release/bioc/html/DESeq2.html
DeltaVision Softworx	GE Healthcare	N/A
Fiji	Schindelin et al., 2012	http://imagej.net/Fiji

CONTACT FOR REAGENT AND RESOURCE SHARING

As Lead Contact, Jane Mellor is responsible for all reagent and resource requests. Please contact Jane Mellor at jane.mellor@bioch.ox.ac.uk with requests and inquiries.

EXPERIMENTAL MODEL AND SUBJECT DETAILS

All yeast strains used in this study are listed in the Key Resources Table. All strains and genetic manipulations were verified by sequencing and phenotype.

Strains were streaked from glycerol stocks onto 2% agar YPD (1% yeast extract (Difco), 1% bactopeptone, 2% glucose) plates and grown (48 hr, 30°C). Cells were then grown (overnight, 30°C) in 5 mL or 25 mL YPD. This culture was used to inoculate a 25 mL or 1 L YPD culture at OD_{600} 0.2 which was grown (30°C, 200 rpm) to OD_{600} 0.6-0.7. For carbon source shift experiments, cell cultures were centrifuged (3000 rpm, 3 min) and then resuspended in YPG (1% yeast extract (Difco), 1% bactopeptone, 2% galactose) prewarmed to 30°C. Resuspended cells were incubated (30°C, 200 rpm) for the specified time.

METHOD DETAIL

Genetic manipulation of yeast strains

All strains used in this study are listed in the KEY RESOURCES TABLE. Genetic manipulation of strains was carried out using the homologous recombination method described by (Longtine et al., 1998). For gene deletion strains, PCR products were made containing a selection marker with promoter and terminator sequences flanked at both ends by 40bp of sequence homologous to sequences either side of the region to be deleted. For 3xFLAG tagged strains, first a plasmid was created from plasmid pFA6a-GFP(S65T)-His3MX6 (Longtine et al., 1998) with the 3xFLAG sequence amplified from strain YSC001 by PCR inserted in place of GFP. PCR products were made using this template consisting of a 40bp sequence homologous to the first 40bp upstream of the stop codon of the gene to be tagged followed by the FLAG sequence, His5 selection marker and 40bp of sequence homologous to a region downstream of the gene to be tagged. 3xHA tagged strains were created in a similar manner using the pFA6a-3HA-KanMX6 plasmid (Longtine et al., 1998).

Cells to be transformed were grown to log phase, pelleted, re-suspended in 450 μL 0.1 M LiAc/TE and incubated (> 1 hr, 4°C). 100 μL of cell suspension, 10 μL of gel-extracted PCR product, 10 μL calf thymus DNA (Sigma D8661), 700 μL 0.1 M LiAc/TE, 40% PEG were incubated (30 min, 30°C) then heat-shocked (20 min, 42°C). Cells were pelleted (5 min, 7000 rpm), re-suspended in H_20 and plated onto appropriate selection media. DNA was extracted from transformants, screened by PCR and confirmed by sequencing.

NET-seq/TEF-seq

Yeast culture. Cells were grown in 25 mL YPD (1% yeast extract (Difco), 1% bactopeptone, 2% glucose) overnight. This was used to inoculate 1 L of YPD in a 2 L flask to give a starting OD_{600} of 0.2. 2 L of cells were grown (30°C, 170 rpm) to OD_{600} of 0.65 (mid-log) and harvested by filtration onto a 0.45 μm pore size, 90 mm diameter nitrocellulose filter paper (Whatman). Cells were scraped off the filter paper with a spatula pre-cooled in liquid nitrogen and flash frozen in liquid nitrogen. Cells were ground in 6 cycles of 3 min at a 15 Hz shaking frequency in a 50 mL grinding jar (Retch) with a 25 mm stainless steel ball using a Retsch MM400 mixer mill. The grinding chamber was cooled in liquid nitrogen in between cycles. 1 g of yeast grindate was stored at −80°C.

For carbon source shift experiments 2 L of cells were grown to mid-log in 2 × 2 L flasks, then centrifuged (3000 rpm, 3 min) and then resuspended in 2 L YPG (1% yeast extract (Difco), 1% bactopeptone, 2% galactose) prewarmed to 30°C. Resuspended cells were returned to 2 L flasks, incubated (30°C, 170 rpm) and harvested by filtration as above after the requisite incubation time.

Immunoprecipitation (IP)

1 g of yeast grindate was resuspended in Lysis buffer A (20 mM HEPES (pH 7.4), 110 mM KOAc, 0.5% Triton X-100, 0.1% Tween 20, 10 mM $MnCl_2$, 1x proteinase inhibitors (complete, EDTA-free (Roche)), 50 U/ml SUPERase.In (Invitrogen)). Resuspended grindate was incubated (4°C, 20 min) with 660 U of DNase I (Promega). Insoluble cell debris was pelleted by spinning (16,000 g, 4°C, 10 min). Supernatants were combined (a 20 μL input sample was taken and combined with 20 μL 2 × SDS loading buffer (80 mM

Tris-HCl (pH 6.8), 200 mM DTT, 3.2% SDS, 0.1% Bromophenol Blue, 1.6% glycerol)) and added to 0.5 mL of anti-FLAG M2 affinity agarose beads (Sigma) pre-washed with 2 × 10 mL Lysis buffer A (without SUPERase.In). Beads and supernatant were incubated (4°C, 2.5 h) on a nutator, before spinning (1,000 g, 4°C, 2 min). A 20 μL unbound sample was taken and combined with 20 μL 2 × SDS loading buffer. Beads were washed four times with 10 mL Wash buffer A (20 mM HEPES (pH 7.4), 110 mM KOAc, 0.5% Triton X-100, 0.1% Tween 20, 1 mM EDTA). Beads were incubated (30 min, 4°C) twice with 300 μL of Elution buffer (Lysis buffer A with 1 mg/mL 3 × FLAG peptide (Sigma)). The elution supernatants were combined (a 20 μL elute sample was taken and combined with 20 μL 2 × SDS loading buffer) and RNAs were extracted using a miRNeasy kit (QIAGEN) according to the manufacturer's instructions. For the sequential IP procedure, the elution supernatants from two IPs were combined giving a total of 1200 ul and added to 15 ul anti-HA dynabeads (Pierce) pre-washed with 2 × 1 mL Lysis buffer A. Beads and supernatant were incubated (4°C, 2.5 h) on a nutator. A 20 μL secondary unbound sample was taken and combined with 20 μL 2 × SDS loading buffer. Beads were washed five times with 1 mL Wash buffer A. Beads were resuspended in 100 uL Lysis buffer A (a 5 uL secondary elute sample was taken and combined with 15 uL Lysis buffer A and 20 uL 2 × SDS loading buffer) and RNAs were extracted using a miRNeasy kit (QIAGEN) according to the manufacturer's instructions. Input, unbound and elute samples were analyzed by western blot.

Western blotting

Protein samples were separated by gel electrophoresis on 7.5% or 10% SDS polyacrylamide gels and transferred to nitrocellulose membranes. Membranes were incubated with 5% BSA in TBST (20 mM Tris-HCl (pH 7.5), 150 mM NaCl, 0.1% Tween-20) for 2 hr, then primary antibody in 2.5% BSA in TBST for 1.5 hr, washed, then incubated with rabbit, mouse or rat HRP-conjugated secondary antibody (Sigma) diluted 1:4000 in 2.5% BSA in TBST for 45 min and washed again. Primary antibodies and their dilutions are detailed in the Key Resources Table. Antibody binding was visualized using chemiluminescence (Pierce) and X-ray film.

Library generation

Linker ligation and RNA fragmentation. Immunoprecipitated RNA (3 μg in 30 μL 10 mM Tris-HCl, pH 7.0) was denatured (2 min, 80°C) and placed on ice. A 5′ adenylated, 3′ blocked with a dideoxy C base cloning linker 5rApp/CTGTAGGCACCATCAAT/3ddC (Integrated DNA Technologies) was ligated to the 3′ ends of RNAs by first dividing the RNA into three microfuge tubes and then adding 10 μL ligation reaction mix to give final concentrations of 50 ng μL^{-1} cloning linker 1, 12% PEG 8000, 1 × T4 RNA ligase 2 (Rnl2) (truncated) ligation buffer, 10 U μL^{-1} T4 Rnl2 (truncated) (NEB) and incubating for 3 hr at 37°C. Ligated RNA was incubated (95°C, 35 min) with 20 μL Alkaline fragmentation buffer (100 mM $NaCO_3$ (pH 9.2), 2 mM EDTA) to fragment linker ligated RNA to a narrow size range to reduce size bias of future steps. RNA was precipitated by incubating (30 min, −20°C) with ice cold 500 μL H_20, 60 μL 3 M NaOAc (pH 5.5), 2 μL 15 mg mL^{-1} GlycoBlue (Ambion) and 0.75 mL isopropanol and then spinning (16,000 g, 4°C, 30 min). Pellets were washed with 0.75 mL 80% ethanol, dried (10 min, room temperature) and resuspended sequentially in the same 10 μL 10 mM Tris-HCl (pH 7.0).

Size selection

Ligated and fragmented RNA was mixed with 10 μL 2 × TBE-urea loading dye (89 mM Tris-HCl, 89 mM Boric acid, 2 mM EDTA, 12% Ficoll, 7 M Urea, 2.5 mg/ml Orange G), denatured (2 min, 80°C) and run on a 10 well 10% TBE-urea gel (Biorad) (200 V, 32 min). The gel was stained with SYBRGold (Invitrogen) and the region containing 38-95nt fragments excised. This gel piece was divided between three microfuge tubes and physically disrupted. Each was incubated (70°C, 10 min) in 200 μL H_20. The tubes were pooled and gel debris was removed using a Costar-Spin-X column (Corning). RNA was precipitated by adding 60 μL 3 M NaOAc (pH 5.5), 2 μL 15 mg mL^{-1} GlycoBlue and 0.75 mL isopropanol, incubating (30 min, −20°C) and then spinning (16,000 g, 4°C, 30 min). Pellets were washed with 0.75 mL 80% ethanol, dried (10 min, room temperature) and resuspended in 10 μL 10 mM Tris-HCl (pH 7.0).

Reverse transcription and circularisation

Reverse transcription (RT) was carried out by adding 3.28 μL 5 × FS buffer, 0.82 μL dNTPs (10 mM each), 0.5 μL 100 μM RT primer (This is phosphorylated at the 5′ end (5 Phos) for later circularization and contains two 18 carbon spacer sequences (iSp18)), denaturing (80°C, 2 min) and incubating (48°C, 30 min) with 0.5 μL Superase.In, 0.82 μL 0.1 M DTT, 0.82 μL Superscript III (Invitrogen). 1.8 μL 1 M NaOH was added and RNA was degraded (98°C, 20 min). To neutralize, 1.8 μL 1 M HCl was added. cDNA was mixed with 20 μL 2 × TBE-urea loading dye, denatured (3 min, 95°C), and run (loaded in 2 wells, 20 μL per well) on a 10 well 10% TBE-urea gel (Biorad) (200 V, 50 min). The gel was stained with SYBRGold (Invitrogen) and the regions containing the RT product excised. Both gel pieces were physically disrupted and incubated (70°C, 10 min) in 200 μL H_20 in separate tubes. Gel debris was removed and tubes were pooled. cDNA was precipitated by adding 25 μL 3 M NaCl, 2 μL GlycoBlue and 0.75 mL isopropanol, incubating (−20°C, 30 min) and then spinning (16,000 g, 4°C, 30 min). The pellet was washed with 0.75 mL 80% ethanol, dried (10 min, room temperature) and resuspended in 15 μL 10 mM Tris-HCl (pH 8.0).

The RT product was circularized by incubating (60°C, 60 min) with 2 μL 10 × CircLigase buffer, 1 μL 1 mM ATP, 1 μL 50 mM $MnCl_2$, 1 μL CircLigase (Epicenter). The enzyme was heat-inactivated at 80°C, 10 min.

PCR amplification

16.7 μL 5 × HF Phusion buffer, 1.7 μL dNTPs (10 mM), 0.4 μL 100 μM Barcoded primer (A, B, C, or D), 0.4 μL 100 μM Primer 1, 59.2 μL H_20, 0.8 μl Phusion polymerase (NEB) was added to 5 μL circularized DNA. This was divided (16.7 μL per tube) between five 0.2 mL tubes. PCR reactions were heated (98°C, 30 s) and then submitted to 7 temperature cycles (98°C, 10 s; 60°C, 10 s; 72°C, 10 s). At the end of cycle 3 and each subsequent cycle one tube was removed and placed on ice. 3.4 μL DNA loading dye (1.5 g Ficoll 400, 25 mg Orange G in 10 mL H_20) was added to each reaction and run on an 8% TBE gel (Invitrogen) (180 V, 55 min). The gel was stained with SYBRGold and the PCR product excised from the PCR reaction with the highest unsaturated signal without higher molecular weight

products. The gel piece was physically disrupted and incubated (room temperature, overnight, with agitation) in 0.67 mL DNA soaking buffer (0.3 M NaCl, 10 mM Tris-HCl pH 8.0, 1 mM EDTA). DNA was precipitated by adding 2 μL GlycoBlue and 0.68 mL isopropanol, incubating (-20°C, 30 min) and spinning (16,000 g, 4°C, 30 min). The pellet was washed with 0.75 mL 80% ethanol, dried (10 min, room temperature) and resuspended in 10 μL 10 mM Tris-HCl (pH 8.0).

Sequencing

Samples were submitted to Harvard Biopolymers facility. They determined DNA quality and quantity using the TapeStation (Agilent) and qPCR and then sequenced samples on an Illumina HiSeq 2000 machine. 50nt were sequenced from one end using the sequencing primer. Barcoded samples were pooled so that 2-3 samples were multiplexed per lane.

See the Key Resources Table for a list of DNA primers used for library generation and sequencing.

Biochemistry

Nuclear Extraction

50 mL from a 75 mL OD_{600} 0.6 culture was pelleted (1,000 g, 3 min), and incubated (< 5 min, 30°C) in 0.5 mL spheroplasting buffer A (1 M sorbitol, 0.5 mM 2-mercaptoethanol, 20 mg/ml zymolase 20T (MP Biomedicals)). Once > 70% of cells were spheroplasted, cells were pelleted (16,000 g, 10 s, 4°C), washed carefully twice with 1 mL 1 M sorbitol, resuspended in 100 μL 18% Ficoll and then mixed quickly with 1 mL 9% Ficoll. Nuclei were pelleted (16,000 g, 5 min, 4°C) and washed twice with 1 mL 1 M sorbitol. RNA was extracted from nuclei as detailed below. Total RNA was extracted from the remaining 25 mL of culture as detailed -below.

RNA extraction

25 mL OD_{600} 0.6 culture was pelleted (1,000 g, 3 min), resuspended in 400 μL T.E.S. (100 mM Tris-HCl (pH 7.5), 100 mM EDTA (pH 8.0), 0.5% SDS) and 400 μL phenol:chloroform (pH 4.7) and incubated (65°C, 20 min, 1400 rpm). After spinning (16,000 g, 20 min, 4°C), the upper layer was mixed with 40 μL 3 M NaOAc pH 5.5 and 1 mL ethanol and incubated (-80°C, 30 min). RNA precipitate was pelleted (16,000 g, 10 min, 4°C) and resuspended in 100 μL H_20.

Reverse transcription

4 μg of extracted RNA was incubated (37°C, 1 h) with 2 μL DNase I buffer, 1 μL RNaseOUT (Invitrogen), 6 μL H_20, 1 μL DNase I (Roche) then heated (70°C, 15 min). 0.5 μg RNA was reverse transcribed by incubating (65°C, 5 min) with 0.6 μL random primers, 1 μL dNTPs in a 13 μL final volume and then adding 4 μL First-Strand buffer, 1 μL 0.1 M DTT, 1 μL RNaseOUT, 1 μL Superscript III (replaced with 1 μL H_20 in control reactions) and incubating at 50°C (1 h) and then 70°C (15 min). Complementary DNA (cDNA) levels for each gene were analyzed by Real Time quantitative PCR (qPCR) using a RotorGene (Corbett), SYBR Green mix (Bioline) and primers. Transcript levels in each sample were normalized to the level of the *ADH1* transcript for the comparison between BY4741 and *paf1Δ* samples as this gene is not Paf1-enriched or depleted in YPD. Transcript levels in each sample were normalized to the level of the *CLB1* transcript for the comparison between BY4741 in YPD and BY4741 after 60 min in YPG samples as the level of Paf1 on Pol2 at this gene does not change between these two conditions. The mean transcript level or nuclear/total transcript level was then calculated across all replicates. Error bars show the SEM. See Table S5 for primers used in qPCR.

Assessingf Paf1 on Pol2 at URA3

We used artificial constructed genes to assess the level of Paf1 on Pol2 downstream of a Paf1-enriched or Paf1-depleted gene promoter. The coding region of the URA3 gene was inserted in place of CMK2 (a Paf1-enriched gene) or SLC1 (a Paf1-depleted gene) coding regions. Thus URA3 was placed downstream of the promoter of a Paf1-enriched or a Paf1-depleted gene. The NET-seq/TEF-seq IP protocol was then carried out on Paf1-3xFLAG, Rpb3-3xFLAG or non-tagged versions of these strains inclusive of the purification of RNA step. 1 μg of purified RNA was reverse transcribed using random primers as above. cDNA levels for 4 control genes (RCR1, TIM21, CLB1 and PUN1) and for test gene URA3 were established for each sample. The level of Paf1, Rpb3 and no-tag signal is expected to be the same in all strains at the control genes. Therefore the control genes were used to determine scaling factors to normalize the signal for each factor to the same level for each strain. An average scaling factor was then determined and this was applied to the signal at URA3. (Paf1 signal - no-tag signal)/(Rpb3 signal - no-tag signal) was calculated for URA3 at each locus to give a value for the level of Paf1 on Pol2. 2 biological replicates were carried out.

RNA fluorescence in situ hybridization (RNA FISH)

35 mL OD_{600} 0.6 culture was fixed by incubating (45 min, 80 rpm, 22°C) with 4.8 mL 32% paraformaldehyde. Fixed cells were washed twice with 10 mL FISH buffer A (1.2 M sorbitol, 0.1 M $KHPO_4$ (pH 7.5)) and resuspended in 1 mL FISH buffer B (FISH buffer A, 20 mM ribonucleoside vanadyl-complex (VRC), 20 μM 2-mercaptoethanol). The mixture was incubated ($\sim$25 min) at 30°C with 15 μL lyticase (25 U μL^{-1}, Sigma) until > 70% of cells were spheroplasted. Cells were pelleted (1000 g, 3 min, 4°C) and washed with and then resuspended in 1 mL FISH buffer B without 2-mercaptoethanol. 150 μL of cells were left to settle (30 min, 4°C) on poly-L-lysine treated coverslips. These were washed with 2 mL FISH buffer A to remove unattached cells, incubated (-20°C, > 3 h) in 2 mL 70% ethanol and then rehydrated for 5 min in 2 mL FISH wash buffer (10% formamide, 2x SSC). For hybridization, coverslips were placed cell-coated side down on a 48 μL drop containing 50 nM of 42 $\times$ 20nt Cy3-labeled probes complementary to *CMK2* (Biosearch Technologies) or 29 $\times$ 20nt Cy3-labeled probes complementary to *GAL1* antisense (AS) transcript, 0.1 g mL^{-1} dextran sulfate, 1 mg mL^{-1} *E. coli* tRNA, 2 mM VRC, 20 μg ml^{-1} BSA, 2x SSC, 10% formamide and incubated (30°C, 20 h) in a sealed Parafilm chamber (See Table S6 for list of FISH probes used). Coverslips were twice incubated (30°C, 30 min) in pre-warmed 2 mL FISH wash buffer, dipped in H_20, air-dried, placed cell-coated side down on a drop of ProLong Diamond Antifade Mountant with DAPI (Life Technologies), allowed to polymerize for 24 hr in the dark and then sealed with nail varnish.

Cells were imaged using a DeltaVision Elite wide-field fluorescence deconvolution microscope using a 100x/1.40 objective lens. For *CMK2*, 31 0.2 μm z stacks were imaged using an exposure time of 0.08 s and 1 s for DAPI and Cy3 channels respectively for each stack. For *GAL1 AS*, 21 0.2 μm z stacks were imaged with an exposure time of 0.01 s and 1 s for DAPI and Cy3 channels respectively.

QUANTIFICATION AND STATISTICAL ANALYSIS

NET-seq/TEF-seq sequencing data processing

Alignment of sequencing reads

FASTQ files with sanger (+33) encoded quality scores were uploaded to Galaxy (Blankenberg et al., 2010). Adaptor sequences were clipped and reads shorter than 15nt after clipping were discarded. Reads were aligned to the *S. cerevisiae* genome (2011 assembly) using Bowtie for Illumina (Langmead, 2010). Parameters were chosen so non-uniquely aligned reads were discarded (-n 1, -e 70, -l 28, -k 1, -m 1). The SAM file output was converted to a BAM file and downloaded (Li et al., 2009).

Narrowing reads and data visualization

Using R/Bioconductor, aligned reads were narrowed to their 3′ nucleotide and used to create BigWig files for data visualization in the IGV (Thorvaldsdóttir et al., 2013). When available, data from combined biological replicates were used for visualization.

Subtraction of non-tag specific signal

Subtraction of non-tag specific signal was done using data from a non-tagged control strain. Signal that is not specific to the FLAG-tagged TEFs, Rpb2 or Rpb3 is present within alignments, mainly resulting from the non-specific binding to and elution from the affinity matrix of processed transcripts undergoing degradation, generating the 3′OH that allows their incorporation into the libraries and alignment. This signal can be removed using signal obtained from a strain grown in the same conditions without a FLAG-tagged factor (no-tag; control IP). To do this the Pol3-transcribed *SCR1* gene (chosen as no signal specific to a factor associated with the Pol2 transcription elongation complex should map to this gene) was divided into 10nt bins exclusive of the first 20nt and final 50nt of the gene (position chrV:442007-442458). The ratio of counts (FLAG-tagged factor/no-tag control) for each bin was calculated. The mean ratio (mean *SCR1* ratio) was taken and mean *SCR1* ratio × no-tag reads were subtracted from FLAG-tagged factor reads genome-wide. For the sequential IP, signal that is not specific to the HA-tagged factor is removed using signal obtained from a strain without an HA-tagged factor (*RPB3-3xFLAG*, no-HA-tag; control IP). In this case the signal that is not specific to the HA-tagged factor results to a greater extent from Pol2 associated nascent transcripts due to non-specific binding of Pol2, enriched during the first IP step, to the HA-beads during the second IP step. Reads over Pol3 transcribed-*SCR1* are not representative of this signal and so cannot be used to determine the ratio necessary for its removal. Instead the ratio of total reads before alignment (HA-tagged factor/no-HA-tag control) was used assuming linear proportionality between this and the signal not specific to the HA-tagged factor. This ratio × no-tag reads were subtracted from HA-tagged factor reads genome-wide.

For visualization, an 11 nt running mean was calculated, except the running mean window did not overlap gene TSSs or TESs or the 3′ nt of exons (upstream of an intron) or 3′ nt of introns. When available, data from combined biological replicates were used for visualization.

mRNA and ncRNA annotations

The TSS and PAS/TES positions of all genes encoding mRNAs, stable lncRNAs (termed SUTs; stable unannotated transcripts) and unstable lncRNAs (termed CUTs; cryptic unstable transcripts) were taken from (Pelechano et al., 2013), selecting first for the most abundant transcript isoforms expressed in YPD and, of these, the longest, to obtain unique annotations for 5579 mRNAs, 612 stable lncRNAs and 344 unstable lncRNAs. The annotations for the start and end of coding sequences, introns, snRNAs, snoRNAs and other ncRNAs were taken from the *Saccharomyces* Genome Database (SGD; http://www.yeastgenome.org/).

Metagene analysis

Data from combined biological replicates when available were used for plotting metagene profiles. All metagene profiles were plotted using single IP TEF-seq and/or single IP NET-seq data unless otherwise stated.

Standard average occupancy profiles

Standard average occupancy profiles (metagene occupancy profiles) were plotted using all mRNA genes unless otherwise stated. For plots of the 5′ region of genes, genes were aligned by their TSS. The final 200nt upstream of the PAS/TES were excluded to avoid transitions occurring at the 3′ end of genes influencing these profiles. The region from the TSS-100nt to TSS+1000nt was split into 10nt bins and the number of counts in each bin was calculated from Rpb3 ChIP-chip data taken from (Mayer et al., 2010) and Rpb3 NET-seq data for wild-type and *paf1Δ* strains. The mean number of counts for each bin at each position was then calculated excluding the top 5% and bottom 5% of bins. These were excluded to prevent highly and lowly transcribed genes from skewing the mean. Mean counts were plotted on a linear scale from the minimum value to the maximum value except otherwise stated. For plots of the 3′ region of genes, genes were aligned by their PAS, genes shorter than 501nt and the region from the TSS to +500nt of genes were excluded to avoid transitions occurring at the 5′ end of genes influencing these profiles. The region from PAS-400nt to PAS+100nt was split into 10nt bins and the mean number of counts for each bin was calculated and plotted in the same manner.

Rpb3-normalized TEF profiles

Rpb3-normalized average TEF occupancy profiles (metagene occupancy profiles) were plotted for all mRNA genes or all lncRNA genes unless otherwise stated. For plots of the 5′ and 3′ end of genes, genes were aligned to the TSS and the PAS respectively

and the same regions were excluded as before. For plots of transitions relative to nucleosomes +2 to +5, the position of nucleosomes was taken from (Brogaard et al., 2012). This publication uses chemical modification of histones to give a base pair resolution genome-wide map of the position of the center of each nucleosome. The region from −100nt to + 100nt around each of the nucleosomes after the TSS+50nt until the PAS were taken from each gene. Again each region was split into 10nt bins and the number of counts from TEF-seq for each TEF divided by the number of counts for Rpb3 NET-seq was calculated. These were multiplied by a factor-specific scaling factor to enable plotting on the same arbitrary linear scale (factor specific scaling factors: Cet1, 1; Paf1, 2.2; Pcf11, 2.7; Spt6, 0.47; Spt16, 1.04; Set2, 6; Ssu72, 8). This is necessary as differences in sequencing depth and the level at which Pol2 is co-immunoprecipitated with different TEFs, leads to large differences in the level of counts for each factor. The mean (scaling factor × TEF)/Rpb3 ratio was then calculated for each bin after first excluding undefined ratios and the top 10% and bottom 10% of ratios. These were excluded to prevent single nucleotide spikes in the data seen with TEF-seq from skewing the mean.

Rpb2-normalized TEF profiles

Rpb2-normalized average TEF occupancy profiles (metagene occupancy profiles) were plotted in the same way as for Rpb3-normalized average TEF occupancy profiles. For single IP data, the mean 0.88 × (Paf1-FLAG TEF-seq)/(Rpb2-FLAG NET-seq) ratio was calculated for each bin. For sequential IP data, the mean 0.75 × (Rpb3-FLAG then Paf1-HA TEF-seq)/(Rpb3-FLAG then Rpb2-HA NET-seq) ratio was calculated for each bin.

Use of Rpb2 or Rpb3 NET-seq data

There are twelve Pol2 subunits in humans and yeast (Rpb1-12 in yeast). Here, single IP NET-seq data, giving a map of transcribing Pol2, have been obtained either by immunoprecipitating Pol2 via its Rpb3 subunit, as in the original protocol (Churchman and Weissman, 2011), or via its Rpb2 subunit. The Pol2 profiles obtained differ slightly depending on which subunit is IP'd, with slightly fewer reads in the 5′ region relative to the 3′ region of genes for Rpb2 than Rpb3. This results in different Pol2-normalized Paf1 occupancy profiles depending on which subunit is used for normalization (see Figures 3B and 3D). We envisage that the more exterior location of the Rpb3 subunit within the Pol2 complex enables this subunit to be more effectively IP'd from all regions of the gene, whereas the Rpb2 subunit, at the center of the Pol2 complex, may be masked by the binding of particular TEFs to Pol2, decreasing the ability to IP it from particular regions. As we predict Rpb3 NET-seq data give a truer signal for the profile of Pol2, we have used these data for the majority of our analysis. Rpb2 NET-seq data have only been used to confirm the similarity between Pol2-normalized Paf1-occupancy profiles obtained from single and sequential IP procedures. This is because only Rpb2 sequential IP NET-seq data have been obtained (Rpb3-FLAG IP followed by Rpb2-HA IP).

Heatmaps

All mRNA genes were ordered by length and split into 10nt bins. Using Rpb3 single IP NET-seq data for wild-type or *paf1Δ* strains, the mean number of counts in each bin was calculated. Negative mean counts were replaced with a zero. Mean counts for each bin across a gene were divided by the sum of the mean counts for that gene to normalize for differences in expression level. These values were converted to a heatmap using a linear scale from white to black.

Differential occupancy analysis

Signal for single IP Paf1 TEF-seq and single IP Rpb3 NET-seq from the TSS+400nt to TES-200nt were counted, thus excluding regions in which Paf1 shows large changes in association with Pol2, for two biological replicates for each carbon source condition. Differential occupancy was assessed using the DESeq algorithm within the DESeq2 package (Love et al., 2014) by comparing the counts for Paf1 and Rpb3 at all mRNA genes (Figure 2A; Table S3) or all mRNA and lncRNA genes (Figure 2B). This gives a $\log_2$ value for the relative Paf1 to Rpb3 occupancy for each gene and a p value, adjusted for multiple testing using the Benjamini-Hochberg method, showing the significance of Paf1-enrichment or depletion on Pol2 at each gene.

For the different downstream analyses carried out on Paf1-enriched and -depleted mRNA gene groups different cut-offs were used based either by selecting genes with $\log_2$ relative Paf1 to Rpb3 occupancy above or below a certain threshold or by selecting genes with an adjusted p value below a certain threshold. Reductions in the stringency of the thresholds used to determine which genes are included in each group, increases the probability of the inclusion of false positives. However, such reductions are necessary for particular analyses to ensure that there are sufficient numbers of genes to analyze. By including larger numbers of genes in particular analyses, any observations are less likely to be the result of random noise thus increasing the robustness of these observations. For metagene profiles, including more genes helps to reduce signal noise. The particular cut-off used in each analysis and the number of genes in each group that this gives is specified when described in the main text.

Divergent promoters enrichment analysis

For the analysis of genes expressed divergently from bi-directional divergent promoters, all yeast mRNA genes at which a $\log_2$ relative Paf1 to Rpb3 occupancy value had been established using the DESeq algorithm were taken. The number of divergently expressed gene pairs within this list was then established (661 gene pairs). Divergently expressed gene pairs were defined as genes transcribed in opposite directions to each other with TSSs within 1200 bp upstream of each other and with no other gene from this list between these two genes. Two groups consisting of the 1200 most Paf1-enriched and the 1200 most Paf1-depleted genes were established based on ordering genes by their $\log_2$ value for relative Paf1 to Rpb3 occupancy. To establish the significance of the enrichment of divergently expressed gene pairs within these groups, 10000 random samples containing 1200 genes were repeatedly taken from the list of genes and the number of pairs within each sample was counted, giving a probability distribution of the number of

expected divergently expressed gene pairs within a random sample of this size. This was compared to the number of divergently expressed gene pairs within each group to give a p value showing the significance of any enrichment or depletion above or below what would be expected by chance.

Promoter motif enrichment analysis

For analysis of the enrichment of motifs within the promoters of Paf1-enriched genes the 500 bp DNA sequence upstream of the TSS was taken from Paf1-enriched genes (p value < 0.1, 229 genes). DREME (Villanyi et al., 2014) was then used to search for significantly enriched motifs on both strands compared to the same promoter regions taken from Paf1-depleted genes (p value < 0.1, 219 genes).

RNA FISH analysis

Images were deconvolved using a conservative deconvolution method and 15 cycles using DeltaVision Softworx software. Image quantification was carried out using Fiji (Schindelin et al., 2012). In this, images were compressed to 2D images displaying the maximum intensity projection for each pixel across z stacks 12 to 22 (for *CMK2*) or 6-16 (for *GAL1 AS*). Cells were outlined manually. For nuclei, an automatic threshold was applied on the DAPI signal using Otsu's thresholding algorithm (Otsu, 1979). Regions corresponding to cell nuclei above the threshold were then automatically outlined. For *CMK2*, Cy3 signal contrast was enhanced by applying the "Sharpen" processing command. Dots corresponding to *CMK2* or *GAL1 AS* transcripts were then counted for cells and nuclei by applying the "Find Maxima" command with a noise tolerance adjusted so that control cells in which *CMK2* or *GAL1* was deleted gave no counts. For *CMK2*, 4 biological replicates were performed for both BY4741 and *paf1Δ* strains with 230-400 cells counted per replicate per strain. For *GAL1 AS*, 3 biological replicates were performed for both $GAL1::ADH1_T$ and $GAL1::ADH1_T$ *paf1Δ* strains with 150-350 cells counted per replicate per strain. The nuclear to whole cell transcript ratio was calculated for each replicate. Bar charts show the mean of these ratios for each transcript in each strain. Error bars show the SEM.

DATA AND SOFTWARE AVAILABILITY

The accession number for the raw and processed sequencing data reported in this paper is ArrayExpress: E-MTAB-4568. Images and other data are available at Mendeley (DOI: 10.17632/2vhybtrwvk.1).

Molecular Cell, Volume *65*

Supplemental Information

Paf1 Has Distinct Roles in Transcription Elongation and Differential Transcript Fate

Harry Fischl, Françoise S. Howe, Andre Furger, and Jane Mellor

SUPPLEMENTAL INFORMATION

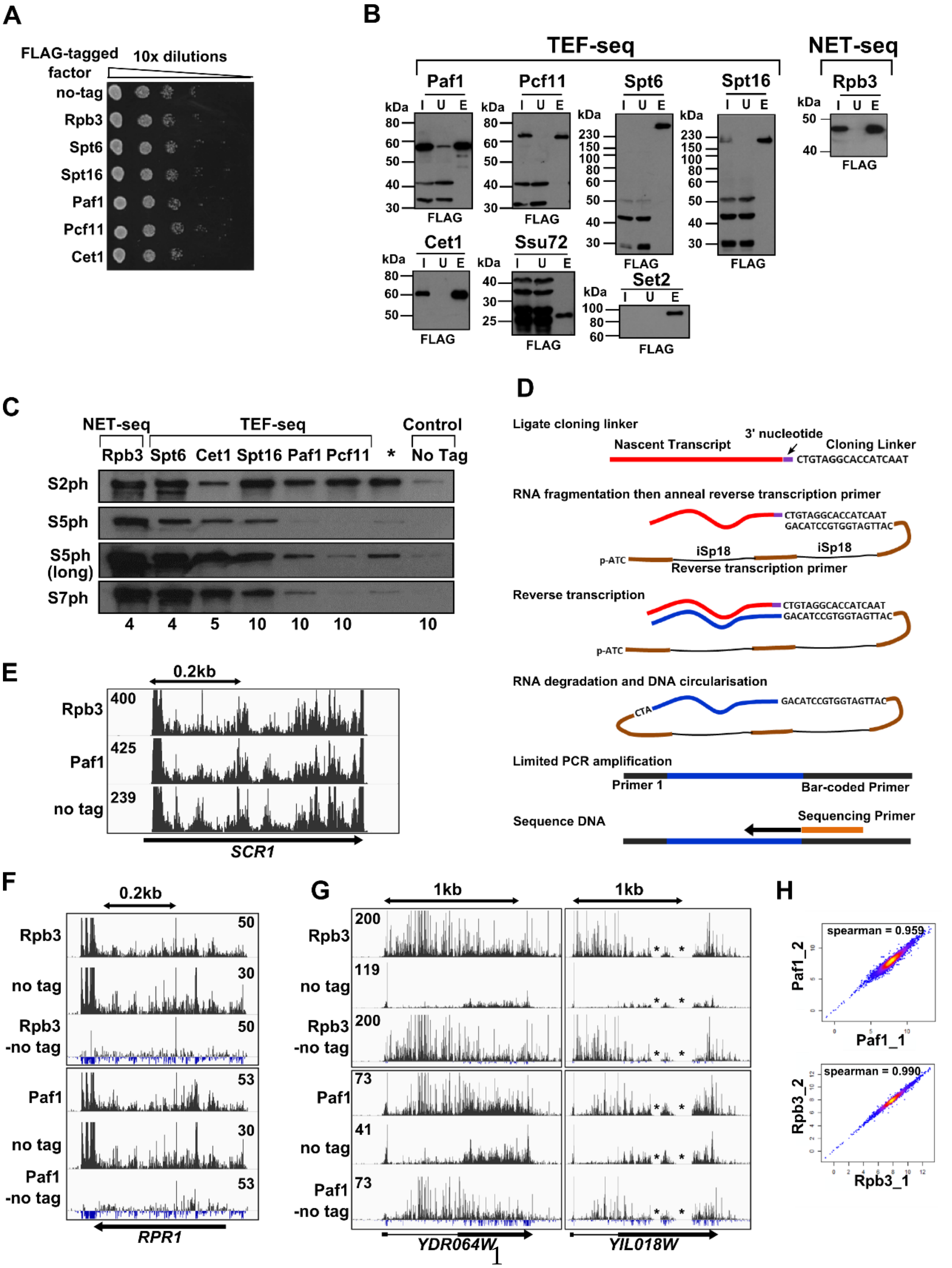

Figure S1, **Related to Figure 1. TEF-seq library preparation and sequencing. A** FLAG-tagging TEFs or Rpb3 does not influence growth rate. Drop plates showing serial 10-fold dilution of strains indicated on rich medium (YPD). **B** Western blots showing IP of FLAG-tagged factors. Large proteins such as Spt6 and Spt16 do not reliably produce a signal in total cell extracts at the concentrations used. I, input; U, unbound; E, eluted with FLAG peptide. **C** Autoradiographs of Western blots showing CTD phosphorylation levels for Ser2, Ser5 (two exposure lengths) and Ser7 on Rpb1 co-IPed with FLAG-tagged Rpb3 (NET-seq), FLAG-tagged TEFs (TEF-seq) or an untagged IP (Control, no-tag). Volume (μl) of each IP used is shown below the autoradiographs. * is an unrelated sample. **D** Schematic of library preparation and sequencing. **E** Background signal that is not specific to the tagged factor, resulting in reads over Pol3-transcribed *SCR1* in raw data from Rpb3 NET-seq, Paf1 TEF-seq and a no-tag control IP (no tag). The level of the signal at *SCR1* was used to remove this background signal from experimental samples genome-wide. **F-G** Images from IGV showing NET-seq reads over Pol2-transcribed *RPS13* and *RPL2B* and Pol3-transcribed *RPR1* for Rpb3-FLAG compared to TEF-seq reads for Paf1-FLAG before (top track) and after (bottom track) subtraction of the no-tag reads from a non-tagged control IP (middle track). * marks gaps in the profiles resulting from discarding non-uniquely aligned reads. Pre-subtraction tracks are scaled relative to the no-tag track according to the ratio of reads in each aligning to the *SCR1* gene. No-tag subtracted tracks are on the same scales as their pre-subtraction tracks. The loss of signal after no-tag subtraction in both the Pol2-specific transcription component Paf1 and Rpb3 tracks at Pol3-transcribed *RPR1* demonstrates that the non-specific signal is effectively removed. **H** Scatter plots showing the high similarity between replicates for NET-seq for Pol2 (Rpb3) and TEF-seq for Paf1. For each replicate, after subtraction of the no-tag control signal, reads mapping to each mRNA gene, from the TSS to the PAS were correlated using spearman correlation coefficients after applying a regularised log (rlog) transformation (Love et al., 2014). Scatter plots are colour-coded from low density (blue) to high density (yellow).(Related to Figure 1).

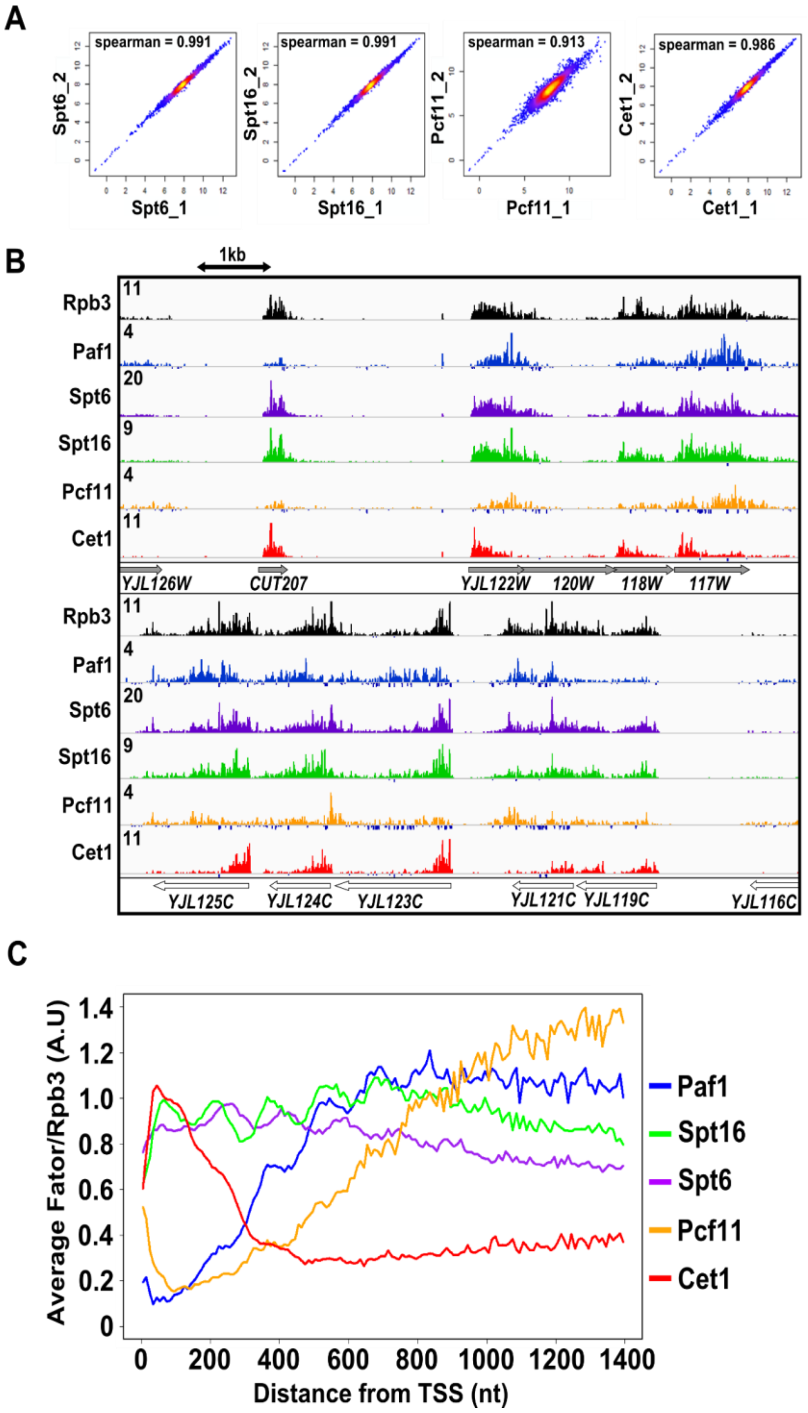
A
spearman = 0.991
Spt6_2
Spt6_1
spearman = 0.991
Spt16_2
Spt16_1
spearman = 0.913
Pcf11_2
Pcf11_1
spearman = 0.986
Cet1_2
Cet1_1
B
1kb
Rpb3
Paf1
Spt6
Spt16
Pcf11
Cet1
YJL126W
CUT207
YJL122W
120W
118W
117W
YJL125C
YJL124C
YJL123C
YJL121C
YJL119C
YJL116C
C
Average Fator/Rpb3 (A.U)
Distance from TSS (nt)
Paf1
Spt16
Spt6
Pcf11
Cet1

29

Figure S2, Related to Figures 2 and 3. TEF-seq for Spt6, Spt16 and Pcf11. **A** Scatter plots showing the high similarity between replicates for TEF-seq for Spt6, Spt16, Pcf11 and Cet1. For each replicate, after subtraction of the no-tag control signal, reads mapping to each mRNA gene, from the TSS to the PAS were correlated using spearman correlation coefficients after applying a regularised log (rlog) transformation (Love et al., 2014). Scatter plots are colour-coded from low density (blue) to high density (yellow). **B** Examples of Rpb3 NET-seq and Paf1, Spt6, Spt16, Pcf11 and Cet1 TEF-seq profiles on individual genes displayed in IGV (see Figure 1 for details). **C** Rpb3-normalized TEF metagene profiles for Paf1, Spt6, Spt16, Pcf11 and Cet1 from the TSS to +1400nt averaged across mRNA genes. See Figure 1**C** for details.

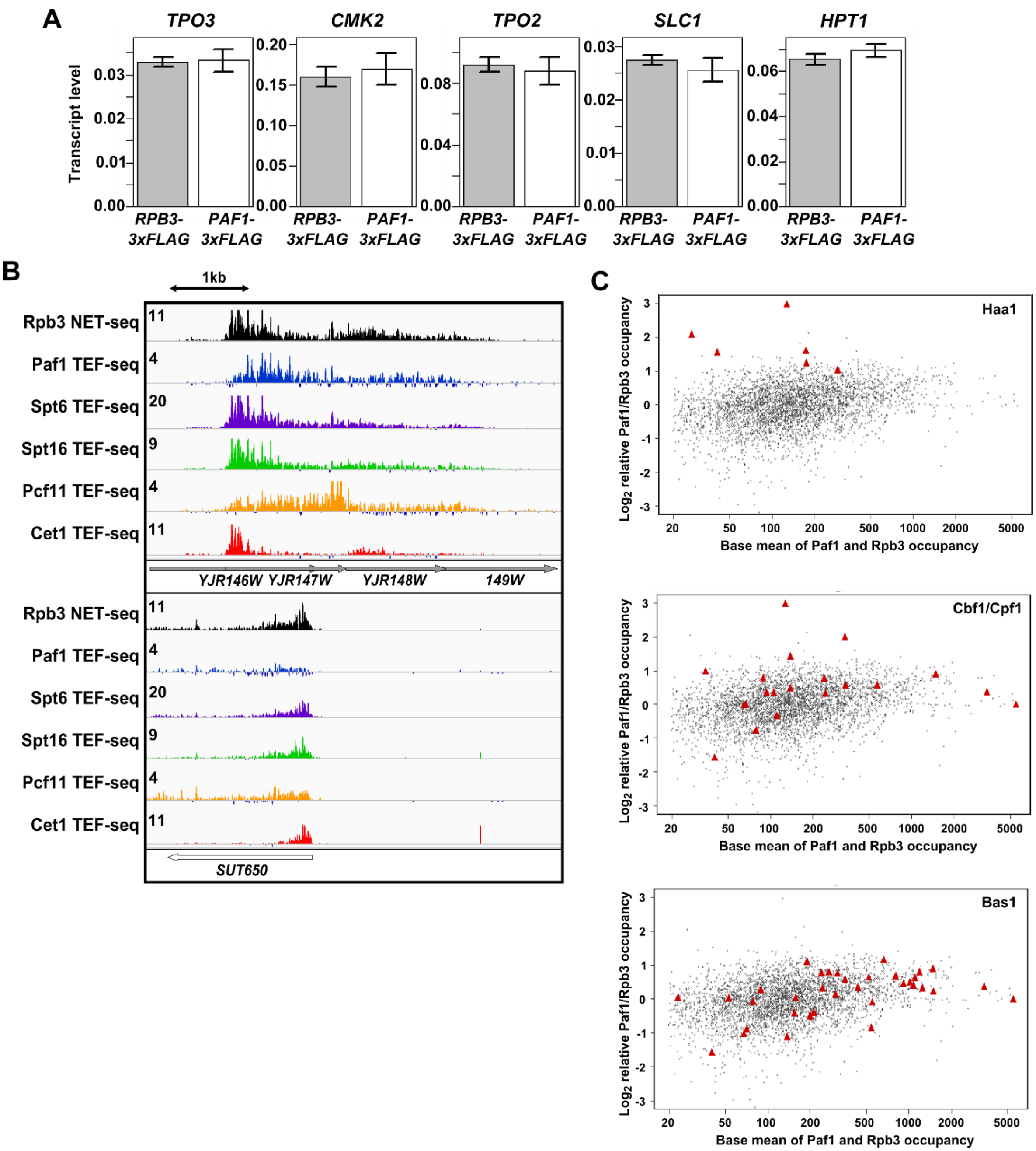
A
TPO3
CMK2
TPO2
SLC1
HPT1
Transcript level
RPB3-3xFLAG
PAF1-3xFLAG
B
1kb
Rpb3 NET-seq
Paf1 TEF-seq
Spt6 TEF-seq
Spt16 TEF-seq
Pcf11 TEF-seq
Cet1 TEF-seq
YJR146W YJR147W
YJR148W
149W
SUT650
C
Haa1
Cbf1/Cpf1
Bas1
Log2 relative Paf1/Rpb3 occupancy
Base mean of Paf1 and Rpb3 occupancy

40

Figure S3, Related to Figure 3. Selective enrichment and depletion for Paf1 on Pol2. A No difference in RNA levels between *PAF1-3xFLAG* and *RPB3-3xFLAG* strains. Bar charts showing the average level of transcripts encoded by Paf1-enriched (*TPO3*, *CMK2*, *TPO2*), Paf1-depleted (*SLC1*) and no significant difference in Paf1 (*HPT1*) genes in *PAF1-3xFLAG* and *RPB3-3xFLAG* strains. Transcript levels, measured by RT-qPCR, are normalized to the level of the *ADH1* transcript in each sample. Error bars show the S.E.M.; n=5. **B** Examples of Rpb3 NET-seq and Paf1, Spt6, Spt16, Pcf11 and Cet1 TEF-seq profiles on individual genes displayed in IGV (see Figure 1 for details). The Paf1 TEF-seq signal is lower relative to the Rpb3 NET-seq signal on the lncRNA gene *SUT650* compared to the mRNA genes *YJR147W* and *YJR148W.* **C** Differential Paf1 occupancy at mRNA genes (grey circles) shown as a scatter plot of the base mean occupancy against the $\log_2$ relative Paf1 to Rpb3 occupancy ratio for each gene. Red triangles show genes regulated by the transcription factors Haa1 (top); Cbf1/Cpf1 (middle) or Bas1 (bottom). Six genes (*YER130C*/*COM2*, *YIR035C*, *YPR156C*/*TPO3*, *YPR157W*/*TDA6*, *YGR138C*/*TPO2, YBR054W/YRO2*) out of 10 that encode Haa1- and acid stress-responsive membrane proteins are shown to be significantly Paf1-enriched. The remaining four genes in this group were too lowly transcribed to be selected for differential occupancy analysis (Keller et al., 2001). Other Paf1-enriched genes, such as *YOL016C*/*CMK2* have also been shown to be regulated by Haa1 (Fernandes et al., 2005; Hu et al., 2007). Like many transcription factors, Bas1 does not show enrichment for regulation of Paf1-enriched genes.

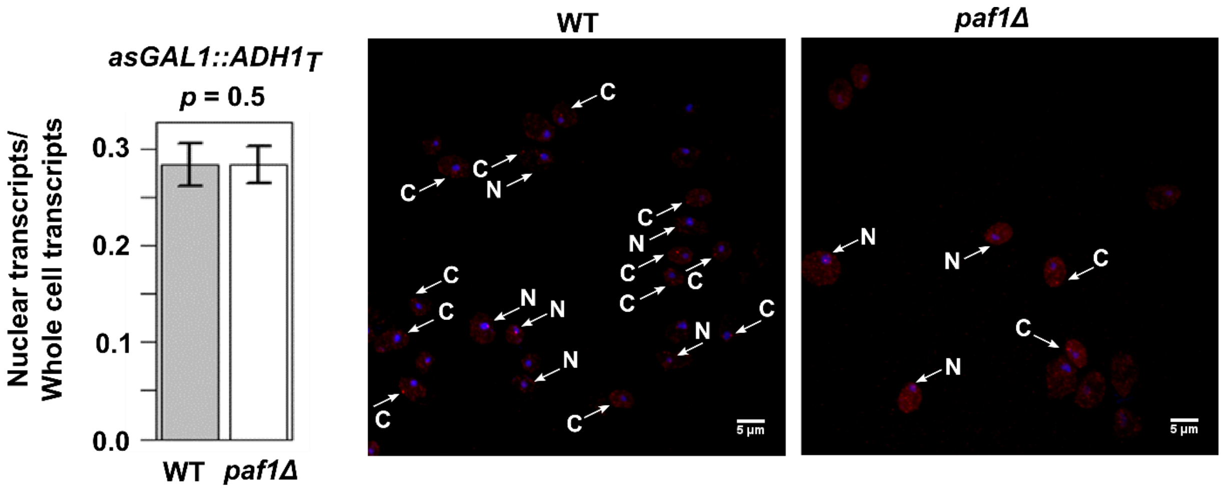

Figure S4

Figure S4, Related to Figure 5. RNA FISH for antisense transcripts at a truncated version of *GAL1* (*GAL1::ADH1*$_T$) in the strains indicated (Murray et al., 2015). Cells were grown in glucose, where the antisense transcript is stably expressed but the galactose inducible *GAL1:ADH1*$_T$ sense transcript is not. Strand-specific probes were used. Bar chart showing the mean ratio of nuclear transcripts/total transcripts. Error bars show the S.E.M., p=0.5, n=3. Representative images showing cells with nuclear (N) or cytoplasmic (C) transcripts used to calculate the ratios in the bar charts.

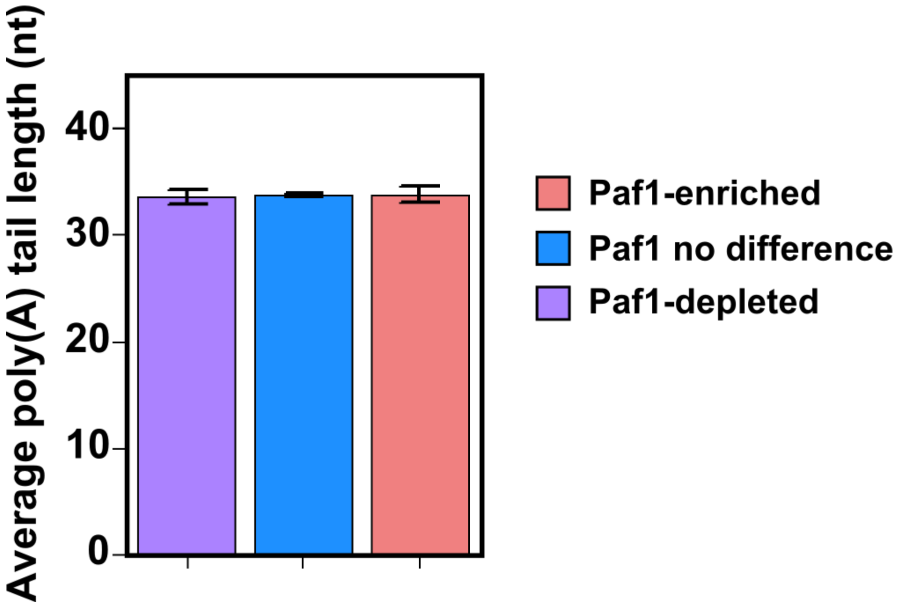

Figure S5, Related to Figures 5 and 6. Differential Paf1 levels do not affect poly(A) tail length. mRNA genes were split into Paf1-enriched (red), Paf1-depleted (purple) and no significant difference in Paf1 (blue) groups ($p < 0.05$). The bar chart shows the mean average poly(A) tail length of transcripts encoded by each group. Error bars show the S.E.M. Transcript poly(A) tail lengths were obtained from Subtelny et al., (2014).

Table S1, Relates to Figure 1-6. Read counts for TEF-seq and NET-seq. Read counts for individual and combined TEF-seq and NET-seq biological replicates before and after subtraction of signal that is not specific to the tagged factor using signal from an untagged control strain (no-tag).

Table S2, Relates to Figures 1-3. Factors subject to TEF-seq.

Table S3, Relates to Figures 4-6. Genes showing enrichment or depletion for Paf1 in yeast cultured in glucose or after 5, 15 and 60 minutes in galactose. Data is shown as log2 FoldChange (log2 relative Paf1 to Rpb3 occupancy ratio). Padj is p value adjusted for multiple testing using the Benjamini-Hochberg method.

Table S4, Relates to Figure 5. PAR-CLIP data analysis. *p* values (Welch's T-test) calculated to test whether there is a significant increase or decrease in the mean average PAR-CLIP factor counts per nucleotide on transcripts encoded by significantly (p value < 0.05) Paf1-enriched or Paf1-depleted mRNA genes, respectively, compared to mRNA genes showing no significant enrichment or depletion for Paf1**.**

Table S5, Relates to Figures 5 and 6. Primers used for qPCR.

**Gene**	**Forward primer sequence**	**Reverse primer sequence**
YPR156C/TPO3	GTGGATCTAAATCCAACATG	ACGGGGGCTTTGGAGCTCAC
YGR138C/TPO2	CTCGTGCAAATCAACTTAAG	GGCGCATTGATATCAGGCAC
YOL016C/CMK2	TGTTCATAGGGATTTGAAAC	CTTCAATTGTTTAGCTATAC
YOL086C/ADH1	GCCACTGACGGTGGTGCTCA	GGCACCAGCTGGCATACCGA

YDR399W/HPT1	GTCGGCAAACGATAAGCAAT	ATGAAACCACCACCACCAAT
YDL052C/SLC1	ATTGACACCTTGAATAAAGG	ACATTGTCAGCTCACTCGTG
YBR005W/RCR1	CAGATGATTATGTTCCTGAG	ATGCAGCCTTATCGTTGGGG
YCR012W/PGK1	GCGTGTCTTCATCAGAGTTG	AGTGAGAAGCCAAGACAACG
YGR033C/TIM21	ACGTCACTGCTTAGAACAAG	TTGAGGCCATAATGGCTTAG
YLR194C/NCW2	CGACATCAACATCAACGGAC	GGCTGAGGACGGTAGCCACC
YLR414C/PUN1	TGACATACTACAACAATTTG	TGATCCAGCGCAATACGTTG
YBR296C/PHO89	TTACTTTAAGTCCACTGATG	ACAGAAACAGTTGTTCCAAG
YGR108W/CLB1	GATGATCCCTTAATGGTGAG	TATGCTTATAGAGATTCGCC
YEL021W/URA3	AGTACTCTGCGGGTGTATAC	TGCCGCCTGCTTCAAACCGC

Table S6, Relates to Figure 5. Probes for RNA FISH.
